# Harnessing Mechanical Stress with Viscoelastic Biomaterials for Periodontal Ligament Regeneration

**DOI:** 10.1002/advs.202309562

**Published:** 2024-03-09

**Authors:** Jiu‐Jiu Zhang, Xuan Li, Yi Tian, Jie‐Kang Zou, Dian Gan, Dao‐Kun Deng, Chen Jiao, Yuan Yin, Bei‐Min Tian, Rui‐Xin Wu, Fa‐Ming Chen, Xiao‐Tao He

**Affiliations:** ^1^ State Key Laboratory of Oral & Maxillofacial Reconstruction and Regeneration, National Clinical Research Center for Oral Diseases, Shaanxi International Joint Research Center for Oral Diseases, Department of Periodontology, School of Stomatology, The Fourth Military Medical University Xi'an 710032 China

**Keywords:** F127 hydrogels, mechanical regulation, periodontal ligaments, viscoelastic hydrogels, viscoelasticity

## Abstract

The viscoelasticity of mechanically sensitive tissues such as periodontal ligaments (PDLs) is key in maintaining mechanical homeostasis. Unfortunately, PDLs easily lose viscoelasticity (e.g., stress relaxation) during periodontitis or dental trauma, which disrupt cell–extracellular matrix (ECM) interactions and accelerates tissue damage. Here, Pluronic F127 diacrylate (F127DA) hydrogels with PDL‐matched stress relaxation rates and high elastic moduli are developed. The hydrogel viscoelasticity is modulated without chemical cross‐linking by controlling precursor concentrations. Under cytomechanical loading, F127DA hydrogels with fast relaxation rates significantly improved the fibrogenic differentiation potential of PDL stem cells (PDLSCs), while cells cultured on F127DA hydrogels with various stress relaxation rates exhibited similar fibrogenic differentiation potentials with limited cell spreading and traction forces under static conditions. Mechanically, faster‐relaxing F127DA hydrogels leveraged cytomechanical loading to activate PDLSC mechanotransduction by upregulating integrin–focal adhesion kinase pathway and thus cytoskeletal rearrangement, reinforcing cell–ECM interactions. In vivo experiments confirm that faster‐relaxing F127DA hydrogels significantly promoted PDL repair and reduced abnormal healing (e.g., root resorption and ankyloses) in delayed replantation of avulsed teeth. This study firstly investigated how matrix nonlinear viscoelasticity influences the fibrogenesis of PDLSCs under mechanical stimuli, and it reveals the underlying mechanobiology, which suggests novel strategies for PDL regeneration.

## Graphical abstract

Viscoelastic F127DA hydrogels with periodontal ligament (PDL)‐matched stress relaxation rates significantly enhance the fibrogenic differentiation of PDL stem cells under cytomechanical loading. Mechanically, viscoelastic F127DA hydrogels harness mechanical stress to reinforce cell–matrix interactions by activating integrin–focal adhesion kinase–cytoskeleton linkages and exhibit potent therapeutic effects on PDL injuries in the delayed replantation of avulsed teeth.

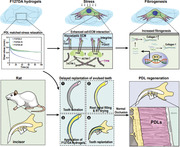

## 1 Introduction

Periodontal ligaments (PDLs) mechanically anchor teeth on the alveolar bone and transfer mechanical loads to adjacent bone.^[^ ^1^, ^2^ ^]^ Due to the hierarchical structure and gradients at soft–hard tissue insertion sites, PDLs have a very high incidence of injury and are susceptible to pathological stimuli, which is the leading cause of tooth loss in adults.^[^ ^3^ ^]^ Globally, 1.1 billion cases of severe periodontitis and 15–61% of cases of dental trauma are associated with PDL injuries.^[^ ^3^, ^4^, ^5^ ^]^ Although many strategies have been developed for reconstructing damaged PDLs, including but not limited to cell sheets,^[^ ^6^, ^7^ ^]^ bioactive scaffolds combined with growth factors or platelet‐rich fibrin,^[^ ^7^, ^8^ ^]^ and enamel matrix derivatives,^[^ ^9^ ^]^ few advances have been achieved due to the complex mechanical microenvironments and hierarchical architectures of PDLs.^[^ ^10^, ^11^, ^12^ ^]^ In the field of periodontal tissue engineering, significant progress has been made in bone regeneration, and guided bone regeneration has been proven to be effective in promoting the healing of both horizontal and vertical bone defects.^[^ ^13^, ^14^ ^]^ However, the therapeutic outcomes of guided tissue regeneration are far from achieving satisfactory regeneration of the bone–PDL–cementum complex.^[^ ^15^ ^]^ Hence, the repair of PDLs may be the main obstacle for the regeneration of the bone–PDL–cementum complex, and finding effective ways to revitalize PDLs can not only benefit the therapy of injured PDLs in dental trauma but also promote the regeneration of the bone–PDL–cementum complex.

PDLs are biomechanically active tissues whose primary function is to bear cyclic mechanical loads and disperse mechanical loads from teeth to the alveolar bone.^[^ ^16^ ^]^ In this mechanically stimulated microenvironment, PDLs have mechanical adaptability and can reshape themselves to maintain their intact structure.^[^ ^17^ ^]^ Under abrupt mechanical loads, the collagen fibers in PDLs can stretch to resist mechanical forces and show time‐dependent responses to mechanical loading or deformation.^[^ ^18^, ^19^ ^]^ These unique biomechanical properties of PDLs are termed viscoelasticity. The viscoelastic properties of PDLs allow the relaxation of traction forces exerted by resident cells on the surrounding extracellular matrix (ECM), enabling the spreading, proliferation, and differentiation of cells in 2D and 3D conditions.^[^ ^20^, ^21^, ^22^ ^]^ Furthermore, the excellent viscoelasticity of PDLs enables them to withstand large mechanical loads and dissipate the excess energy, which can prevent the detrimental effects of excessive mechanical loads on embedded cells.^[^ ^23^, ^24^, ^25^ ^]^ However, the viscoelasticity of PDLs can be easily lost due to periodontitis or dental trauma, compromising the biomechanical function of the periodontium.^[^ ^16^ ^]^ Under such circumstances, the cell–ECM interactions change, and even physiological mechanical loads can lead to periodontal tissue damage. Therefore, recovering the mechanical adaptability of the periodontium by restoring the biomechanical properties of PDLs (e.g., viscoelasticity) is a novel and promising way to promote periodontal regeneration.

Pluronic F127 diacrylate (F127DA) hydrogels are nanomicelle crosslinked hydrogels with excellent viscoelasticity that can structurally and biomechanically mimic PDLs.^[^ ^26^ ^]^ Moreover, F127DA hydrogels have been widely used in drug delivery for periodontal regeneration.^[^ ^27^, ^28^ ^]^ Therefore, F127DA hydrogels were chosen for fabricating viscoelastic biomaterials. By controlling the precursor concentrations, F127DA hydrogels with tunable elastic modulus and stress relaxation were successfully synthesized. Then, the effects of viscoelastic F127DA hydrogels on the fibrogenic differentiation of PDL stem cells (PDLSCs) were investigated under static conditions and under cytomechanical loading. Given that mechanotransduction determines how cells translate extracellular mechanical stimuli to intracellular biochemical signals,^[^ ^29^ ^]^ the effects of F127DA hydrogels on the activation of integrin–focal adhesion kinase (FAK) signaling and cytoskeletal rearrangement were further evaluated under cytomechanical loading. Finally, the repair‐promoting functions of viscoelastic F127DA hydrogels were tested in the delayed replantation of avulsed teeth. These findings, for the first time, have elucidated how hydrogels with stress relaxation determine cellular behaviors under cytomechanical loading and have suggested a new strategy to promote the regeneration of PDLs.

## 2 Results and Discussion

### 2.1 The Mechanical and Rheological Characterization of F127DA Hydrogels

**Figure 1:**
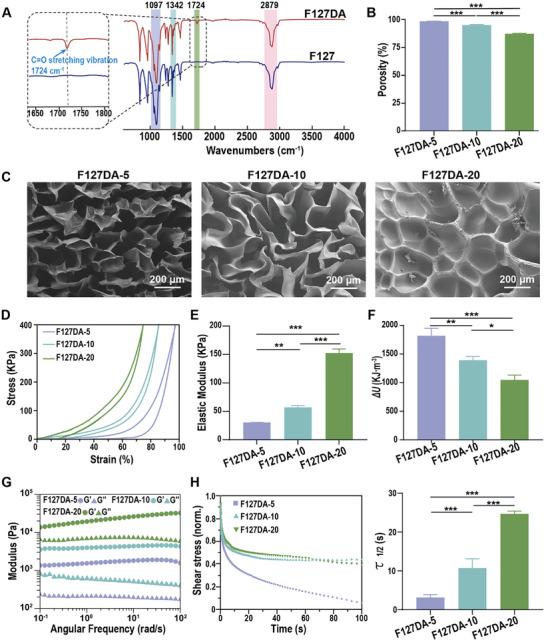
Characterization of viscoelastic F127DA‐5, F127‐10 and F127‐20 hydrogels. A) FT‐IR spectra of F127 and F127DA. The left panel showing the magnified images of characteristic peak of C═O stretching vibration at 1724 cm^−1^. B) The porosity of F127DA‐5, F127‐10, and F127‐20 hydrogels (*n* = 4). C) Representative SEM images showing the microstructures of F127DA‐5, F127DA‐10, and F127DA‐20 hydrogels. Scale bar = 200 µm. D) Cyclic loading‒unloading compressive curves of F127DA‐5, F127DA‐10 and F127DA‐20 hydrogels. E) Initial elastic modulus of F127DA hydrogels as calculated from compression curves of viscoelastic F127DA‐5, F127DA‐10, and F127DA‐20 hydrogels (*n* = 3). F) Energy dissipation of F127DA hydrogels determined by compression testing of viscoelastic F127DA‐5, F127DA‐10, and F127DA‐20 hydrogels (*n* = 3). G) Frequency dependency of storage modulus G′ and loss modulus (G″) for F127DA‐5, F127DA‐10, and F127DA‐20 hydrogels. H) Normalized stress relaxation tests on viscoelastic F127DA hydrogels at 1% strain and quantification of the half stress‐relaxation time (*τ*_1/2_) determined by stress relaxation testing of F127DA hydrogels (*n* = 3). The data are shown as the mean ± SD. Data shown in (B), (E), (F), and (H) were analyzed by one‐way ANOVA; *p < 0.05, **p < 0.01 and ***p < 0.001 indicate significant differences between the indicated columns.

In the present study, we fabricated F127DA hydrogels to mimic the viscoelastic properties of PDLs. The reason for this choice is that F127DA hydrogels can self‐assemble into nanomicelles that closely resemble the nanofibers of PDLs through photoinitiated free radical copolymerization.^[^ ^26^, ^30^ ^]^ Moreover, the F127DA hydrogels exhibit minimal degradation due to the main covalent intramicelle bridging and adjacent intermicelle crosslinks in the networks, which can offer durable and consistent physical and chemical characteristics for investigating the effects of viscoelastic properties on cells.^[^ ^26^ ^]^ Furthermore, F127DA hydrogels are highly versatile, with tunable viscoelasticity and other excellent physiochemical properties, such as low swelling and high strength, which are also quite suitable for PDL repair.^[^ ^31^, ^32^ ^]^ First, the successful synthesis of F127DA was confirmed by Fourier transform infrared spectroscopy (FT‐IR). As shown in **Figure** **1**A, it was found that both F127 and F127DA displayed the representative absorbance peaks of C─O stretching at 1097 cm^−1^, in‐plane O─H bending at 1342 cm^−1^, and C─H aliphatic stretching at 2879 cm^−1^. However, the characteristic peak of C═O stretching vibration at 1724 cm^−1^ was only evident in the FT‐IR spectrum of F127DA, indicating that F127DA was successfully synthesized by grafting a vinyl group onto F127 through a condensation reaction. Then the porosity, microstructure, elastic modulus, and viscoelasticity of F127DA hydrogels with different concentrations were evaluated by the liquid displacement method, scanning electron microscopy (SEM), cyclic loading‒unloading compressive tests, and rheological tests. As shown in Figure 1B, the porosity of F127DA hydrogels decreased with the concentration; and the porosity of the F127DA‐5, F127DA‐10, and F127DA‐20 hydrogels was 96.16 ± 0.43%, 92.91 ± 0.78%, and 85.23 ± 0.57%, respectively. Representative SEM images showed irregular porous microstructures in all the tested hydrogels, and the wall thickness of the F127DA hydrogels increased with the precursor concentration (Figure 1C). The cyclic loading‒unloading compressive tests showed that the stress–strain curves of the F127DA‐5, F127DA‐10, and F127DA‐20 hydrogels were nonlinear (Figure 1D). As the precursor concentration of the F127DA hydrogels increased from 5% to 10% to 20% (wt/vol), the elastic modulus of the F127DA hydrogels changed from 29.91 ± 0.73 KPa to 56.64 ± 3.65 KPa and then reached a maximum of 152.08 ± 7.65 KPa (Figure 1E). Notably, there were significant differences in the hysteresis loops of each hydrogel during the unloading period, indicating the different capacities of the F127DA‐5, F127DA‐10, and F127DA‐20 hydrogels to dissipate energy.^[^ ^4^, ^33^ ^]^ Quantitative analysis showed that the F127DA‐5 hydrogels exhibited the highest dissipation energy (1813 ± 135.37 J m^−3^) among all tested hydrogels (Figure 1F). Furthermore, the mechanical stability of F127DA hydrogels was analyzed. As shown in Figure S1, Supporting Information, the loading–unloading curves from 5 cyclic compressive tests were consistent across the tested groups, suggesting that the mechanical characteristics of F127DA‐5, F127DA‐10, and F127DA‐20 hydrogels remained stable. Subsequently, the viscoelastic properties of hydrogels were analyzed in terms of rheological properties. Frequency sweeps from 0.1 to 100 rad s^−1^ were conducted at 37 °C to determine the viscoelasticity of the preprepared F127DA hydrogels, and the strain was set to 1% to ensure that the entire oscillatory deformation test was conducted within the linear elastic regime of the hydrogels. The results showed that the storage modulus (G′) was larger than the loss modulus (G″) and changed only slightly over the entire frequency range in all the hydrogels, indicating the gel‐like behavior of F127DA hydrogels, and both G′ and G″ increased with the precursor concentration (Figure 1G). To investigate the stress relaxation rate of the F127DA hydrogels, a constant 1% strain was applied, and the stress in response to strain was measured over the course of 100 s. The results indicated that, compared to the F127DA‐10 and F127DA‐20 hydrogels, the F127DA‐5 hydrogels exhibited the fastest stress relaxation rate. Consistently, *τ*_1/2_ (the time over which the initial stress was relaxed to half its value) decreased from 24.69 ± 0.5 s to 10.77 ± 2.37 s and reached a minimum of 3.17 ± 0.1 s when the concentrations decreased from 20% to 10% to 5% (wt/vol) (Figure 1H).

The hierarchical composites of ECM in PDLs containing self‐assembled collagen fibers and noncovalently crosslinked macromolecules (e.g., glycoproteins or polysaccharides) allow the rearrangement of fibers and the reformation of weak bonds, serving as the primary source of tissue viscoelasticity.^[^ ^34^, ^35^ ^]^ Despite great progress in biomaterials science,^[^ ^36^, ^37^ ^]^ it is still impossible to recapitulate the viscoelastic properties of PDLs due to the complex interconnected networks of ECM. Inspired by the self‐assembly and dynamic crosslinking principles of native ECM, increased efforts have been devoted to incorporating reversible crosslinks (e.g., reversible supramolecular interactions or noncovalent chemical reactions) into networks of synthetic biomaterials for engineering applications.^[^ ^38^, ^39^ ^]^ However, these modulations result in biomaterials with slower stress relaxation,^[^ ^40^ ^]^ which still cannot mimic the excellent viscoelasticity of PDLs. Moreover, the low elastic moduli of viscoelastic materials, such as alginate hydrogels (≈10 KPa),^[^ ^21^ ^]^ borate ester bond‐modified gelatine methacryloyl hydrogels (≈8 KPa),^[^ ^41^ ^]^ boronate‐based hydrogels (≈2 KPa),^[^ ^42^ ^]^ collagen hydrogels (≈0.3 KPa)^[^ ^43^ ^]^ and protein‐engineered hyalurona hydrogels (≈1 KPa),^[^ ^44^ ^]^ limits their use in the regeneration of tissues under dynamic mechanical loading. In the present study, we provided an easy and versatile way to fabricate viscoelastic hydrogels with tailored stress relaxation and high elastic moduli (Figure 1). By varying the concentration of F127DA, both the stress relaxation and the elastic modulus could be precisely controlled. The *τ*_1/2_ of F127DA hydrogels can reach ≈3.17 ± 0.1–24.69 ± 0.5 s, which is much better than that of frequently used alginate hydrogels.^[^ ^21^ ^]^ The fast stress relaxation of F127DA hydrogels also closely matched the stress relaxation of PDLs, whose *τ*_1/2_ varies from seconds to tens of seconds due to the heterogeneity of PDLs.^[^ ^12^, ^23^ ^]^ The excellent energy dissipation or fast stress relaxation of F127DA hydrogels is due to the deformation of macromolecular micelles or the disentanglement of polymer chains with increasing water content.^[^ ^26^ ^]^ Moreover, the elastic modulus of the F127DA hydrogels ranged from 29.91 ± 0.73 KPa to 152.08 ± 7.65 KPa, which is significantly higher than the elastic modulus of most viscoelastic hydrogels.^[^ ^21^, ^41^, ^42^, ^43^ ^]^ Based on these aforementioned findings, we believe that the obtained F127DA hydrogels might be suitable for PDL repair due to their favorable mechanical properties.

### 2.2 The Cytocompatibility and Histocompatibility of F127DA‐5, F127DA‐10, and F127DA‐20 Hydrogels

**Figure 2:**
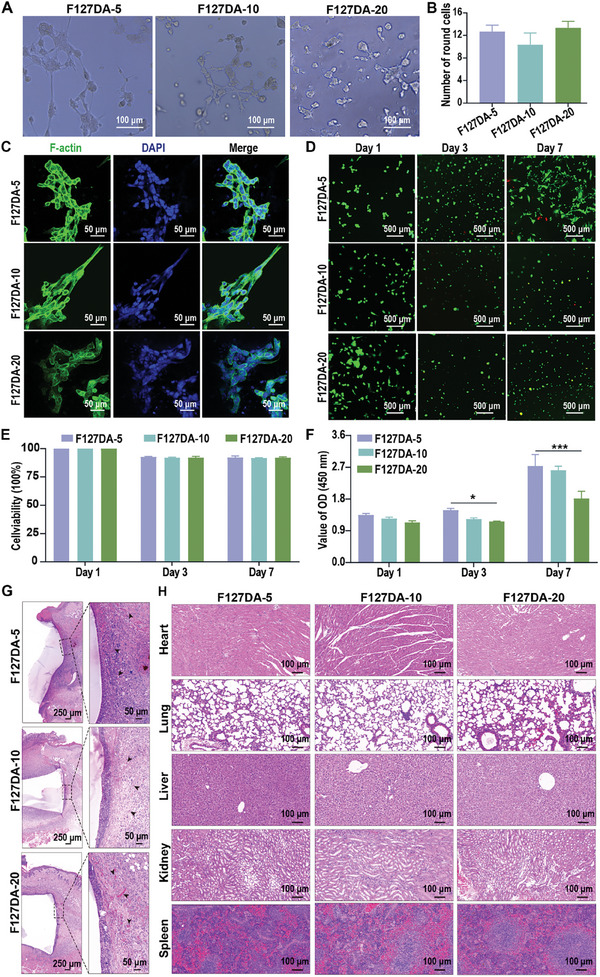
The biocompatibility of F127DA‐5, F127DA‐10 and F127DA‐20 hydrogels. A) Representative optical microscope images showing the morphology of PDLSCs cultured on the surfaces of F127DA‐5, F127DA‐10, and F127DA‐20 hydrogels. Scale bar = 100 µm. B) Quantitative analysis of nonspread round cells cultured on F127DA‐5, F127DA‐10, and F127DA‐20 hydrogels (*n* = 3). C) Representative images of phalloidin staining showing the cytoskeletons of PDLSCs cultured on the F127DA‐5, F127DA‐10, and F127DA‐20 hydrogels for 24 h. Scale bar = 50 µm. D) Representative images of live/dead cell staining of PDLSCs cultured on F127DA‐5, F127DA‐10, and F127DA‐20 hydrogels on days 1, 3, and 7. Scale bar = 500 µm. E) Quantitative analysis of cell viability determined by live/dead cell staining (*n* = 3). F) CCK‐8 assays showing the proliferation of PDLSCs cultured on F127DA‐5, F127DA‐10, and F127DA‐20 hydrogels on days 1, 3, and 7 (*n* = 3). G) The representative images of H&E staining showing tissues surrounding the implanted hydrogels at 2 weeks post surgery. Left panels, the overview of the hydrogel‐implanted area, scale bar = 250 µm; right panel, the magnified view focusing on infiltrated immune cells and newly formed blood vessels, scale bar = 50 µm. H) Representative images of H&E staining for major organs of rats receiving F127DA‐5, F127DA‐10, and F127DA‐20 hydrogels, scale bar = 100 µm. The data are shown as the mean ± SD. Data shown in (B) were analyzed by one‐way ANOVA. Data shown in (E) and (F) were analyzed by two‐way ANOVA; *p < 0.05 and ***p < 0.001 indicate significant differences between the indicated columns.

The cytocompatibility of F127DA hydrogels with PDLSCs was first evaluated by examining the adhesion, morphology, and viability of PDLSCs on F127DA‐5, F127DA‐10, and F127DA‐20 hydrogels. Representative optical microscopy images showed that PDLSCs could adhere to the surface of arginine‐glycine‐aspartic (RGD)‐modified F127DA hydrogels. Additionally, most cells cultured on the F127DA‐5, F127DA‐10, and F127DA‐20 hydrogels had uniform round and compact cell shapes (**Figure** **2**A). The quantitative analysis revealed no significant differences in the number of round cells cultured on the F127DA hydrogels (Figure 2B). Consistently, the representative images of phalloidin staining showed that PDLSCs cultured on the F127DA‐5, F127DA‐10, and F127DA‐20 hydrogels had similar cellular morphology (Figure 2C). Furthermore, the viability of PDLSCs cultured on F127DA‐5, F127DA‐10, and F127DA‐20 hydrogels was assessed by live/dead cell staining and cell counting kit‐8 (CCK‐8) assays (Figure 2D–F). On days 1, 3, and 7 of incubation, representative images of live/dead cell staining showed that most cells cultured on all the F127DA hydrogels were living (labeled with green fluorescence) (Figure 2D). In line with the live/dead staining, quantitative analysis showed that the percentages of living cells (>90%) in different groups were similar on days 1, 3, and 7 of incubation, suggesting that F127DA hydrogels support excellent cell viability (Figure 2E). The CCK‐8 assay indicated that cells cultured on F127DA‐5, F127DA‐10, and F127DA‐20 hydrogels showed a trend toward increasing proliferation, and the optical density (OD) value of cells cultured on F127DA‐5 hydrogels was higher than that of cells cultured on F127DA‐20 hydrogels on days 3 and 7 during incubation (Figure 2F). Altogether, these data indicated good cytocompatibility of the F127DA hydrogels. To further confirm the biocompatibility of F127DA hydrogels, F127DA‐5, F127DA‐10, and F127DA‐20 hydrogels were subcutaneously implanted into the dorsal flanks of mice. During the whole experiment period, none of the surgical sites showed any sign of infection and all animals survived in the experiment. The representative images of hematoxylin and eosin (H&E) staining showed the presence of immune cells surrounding the implanted F127DA‐5, F127DA‐10, and F127DA‐20 hydrogels, although no fibrous tissues could be observed as 2 weeks post surgery. The enlarged area showed many newly formed blood vessels (black arrows) in the transplanted area (Figure 2G). In addition, no signs of any tissue damage (e.g., inflammation, hemorrhage, necrosis) could be observed in the major organs (e.g., heart, lung, liver, kidney, and spleen) (Figure 2H). Combined with the in vitro data, these findings indicated that F127DA hydrogels are non‐toxic.

Previously published studies have indicated that the spreading area of cells increases with the substrate modulus or stress relaxation rates of the matrix due to modification of their mechanical response.^[^ ^45^, ^46^, ^47^ ^]^ However, we found that differences in the stress relaxation/elastic moduli of the F127DA hydrogels exerted little effect on cell morphology (Figure 2), as cells cultured on F127DA‐5, F127DA‐10 or F127DA‐20 hydrogels all displayed limited cell spreading, indicating the uncoupling of the nucleus and the actin cytoskeleton (Figure 2). The reason may be that F127DA hydrogels with fast stress relaxation exhibited a decreased elastic modulus (Figure 1). Therefore, the increased stress relaxation‐induced cell spreading might compensate for the low elastic moduli of F127DA hydrogels, which is consistent with previously published studies.^[^ ^48^ ^]^ Furthermore, the atomic force microscopy (AFM) analysis indicated similar cell elastic moduli among cells cultured on F127DA‐5, F127DA‐10, and F127DA‐20 hydrogels, implying that cells in different groups exerted similar traction forces on the ECM (Figure S2, Supporting Information). Considering that cells sense the stiffness and stress relaxation of ECM mainly by gauging resistance to the traction forces they exert on the matrix,^[^ ^21^ ^]^ these data suggested that F127DA hydrogels with different viscoelasticities/elastic moduli exerted similar mechanical regulation on cells.

### 2.3 Viscoelastic F127DA Hydrogels Enhanced the Fibrogenic Differentiation Potential of PDLSCs under Cytomechanical Loading

**Figure 3:**
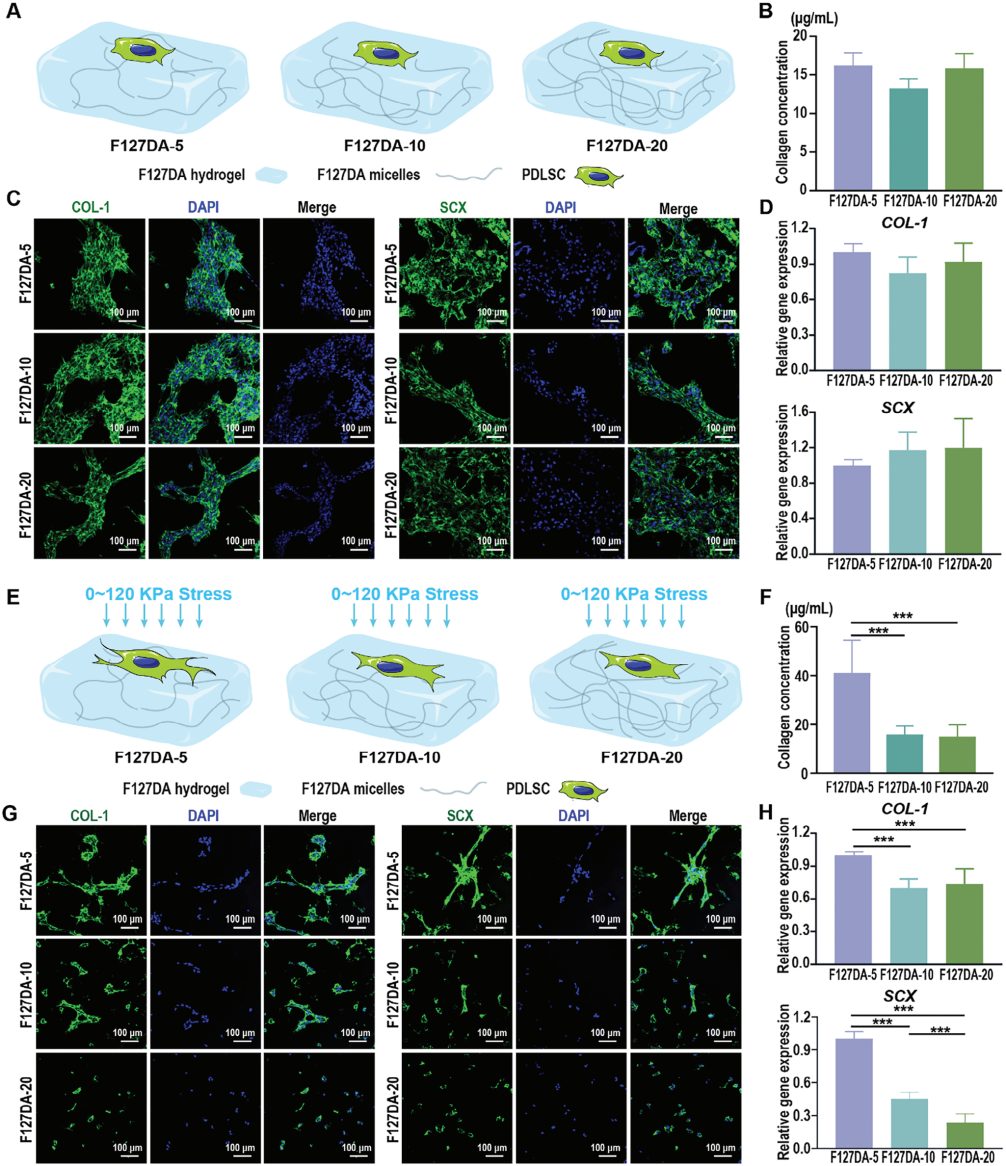
The fibrogenic differentiation potentials of PDLSCs cultured on viscoelastic F127DA hydrogels. A) Schematic diagrams of PDLSCs cultured on viscoelastic F127DA hydrogels under static conditions. B) The concentrations of total collagen produced by PDLSCs cultured on F127DA‐5, F127DA‐10, and F127DA‐20 hydrogels under static conditions for 7 days (*n* = 6) (Sirius red total collagen detection assays). C) Representative images of immunofluorescence staining showing the fibrogenic differentiation‐related markers (COL‐1 and SCX) of PDLSCs cultured on F127DA‐5, F127DA‐10, and F127DA‐20 hydrogels under static conditions for 7 days. D) Expression of fibrogenic differentiation‐related genes (*COL‐1* and *SCX*) in PDLSCs cultured on F127DA‐5, F127DA‐10, and F127DA‐20 hydrogels under static conditions for 7 days (*n* = 6) (qRT–PCR assay). E) Schematic diagrams of PDLSCs cultured on viscoelastic F127DA hydrogels under cytomechanical loading. F) The concentrations of total collagen produced by PDLSCs cultured on F127DA‐5, F127DA‐10, and F127DA‐20 hydrogels for 7 days under cytomechanical loading (≈0–120 KPa, 1 Hz, 1 h day^−1^) every other day (*n* = 6) (Sirius red total collagen detection assays). G) Representative images of immunofluorescence staining showing the fibrogenic differentiation‐related markers (COL‐1 and SCX) of PDLSCs cultured on the F127DA‐5, F127DA‐10, and F127DA‐20 hydrogels under cytomechanical loading. H) Expression of fibrogenic differentiation‐related genes (*COL‐1* and *SCX*) in PDLSCs cultured on the F127DA‐5, F127DA‐10, and F127DA‐20 hydrogels under cytomechanical loading (*n* = 6) (qRT–PCR assay). The data are shown as the mean ± SD; Data shown in (B), (D), (F) and (H) were analyzed by one‐way ANOVA; ***p < 0.001 indicates significant differences between the indicated columns.

The viscoelasticity of PDLs allows time‐dependent energy dissipation, which plays a key role in maintaining mechanical homeostasis in periodontal tissues by decreasing internal stress over time in response to step deformation (termed “stress relaxation”). Recently, the importance of stress relaxation in cell–ECM interactions has gained extensive attraction. Mooney et al. showed that hydrogels with fast relaxation can significantly increase stem cell spreading and osteogenic differentiation,^[^ ^21^, ^34^, ^48^ ^]^ determine the dynamics of tissue growth,^[^ ^22^ ^]^ and even regulate the immune response of immune cells.^[^ ^49^, ^50^ ^]^ These milestone studies highlight hydrogel viscoelasticity as a key design parameter for cell fate decisions. However, whether and how viscoelasticity, especially stress relaxation, influences cell behaviors under external stress loading is still unknown. Considering that mechanical stress is inevitable in periodontal tissues and is necessary for PDL repair,^[^ ^51^ ^]^ we further explored whether viscoelastic F127DA hydrogels can increase the fibrogenic differentiation potential of PDLSCs under cytomechanical loading. The fibrogenic differentiation potentials of PDLSCs cultured on viscoelastic F127DA hydrogels were first evaluated by Sirius red total collagen detection, quantitative real‐time polymerase chain reaction (qRT–PCR), and immunofluorescence staining, respectively. First, the influences of the F127DA‐5, F127DA‐10, and F127DA‐20 hydrogels on the fibrogenic differentiation potential of PDLSCs under static conditions were evaluated (**Figure** **3**A). After 7 days of incubation, Sirius red total collagen detection showed no significant differences in the content of soluble collagen generated by PDLSCs cultured on different hydrogels under static conditions (Figure 3B). Consistently, representative images of immunofluorescence staining showed that PDLSCs cultured on the F127DA‐5, F127DA‐10, and F127DA‐20 hydrogels exhibited comparable expression levels of fibrogenic differentiation‐related markers (collagen type‐1 (COL‐1) and scleraxis (SCX)) (Figure 3C). In line with these results, no significant difference could be observed in the expression of fibrogenic differentiation‐related genes (*COL‐1* and *SCX*) in PDLSCs among all tested groups (Figure 3D). Collectively, these data suggest that under static conditions, the F127DA‐5, F127DA‐10, and F127DA‐20 hydrogels with different stress relaxations and elastic moduli exerted similar influences on the fibrogenic differentiation potential of PDLSCs. To mimic the hydrostatic pressure endured by cells encapsulated in interstitial fluid‐filled ECM during mastication,^[^ ^4^, ^52^, ^53^ ^]^ dynamic compressive stress (≈0–120 KPa, 1 Hz, 1 h day^−1^) was applied to PDLSCs cultured on F127DA‐5, F127DA‐10 and F127DA‐20 hydrogels (Figure 3E). Notably, F127DA‐5 hydrogels significantly increased the collagen production of PDLSCs under cytomechanical loading compared with those grown on F127DA‐10 or F127DA‐20 hydrogels (Figure 3F). Representative images of immunofluorescence staining also revealed that fibrogenic differentiation‐related markers (COL‐1 and SCX) were more abundant in PDLSCs cultured on the F127DA‐5 hydrogels than in PDLSCs cultured on the F127DA‐10 or F127DA‐20 hydrogels under cytomechanical loading (Figure 3G). Consistent with immunofluorescence staining, qRT–PCR analysis revealed that PDLSCs cultured on viscoelastic F127DA‐5 hydrogels exhibited higher expression levels of fibrogenic differentiation‐related genes (*COL‐1* and *SCX*) than cells cultured on F127DA‐10 and F127DA‐20 hydrogels when subjected to dynamic compressive stress (Figure 3H). Taken together, our results indicate that under external cytomechanical loading, F127DA‐5 hydrogels with faster stress relaxation rates and lower elastic moduli can promote the fibrogenic differentiation of PDLSCs.

Although the roles of nonlinear viscoelasticity in determining cell–ECM interactions have been widely recognized, most of these studies focus on how cells respond to the stress–relaxing matrix without external mechanical stimuli, and the results of such studies are often assumed to predict the mechanical environment experienced by cells in vivo.^[^ ^21^, ^43^, ^54^ ^]^ However, tissues and ECM are often under dynamic external mechanical loading (e.g., PDL), which is an important regulator in maintaining physiological function maintenance.^[^ ^55^, ^56^ ^]^ For example, mechanical force has been reported to promote type H angiogenesis and osterix+ (OSX+) cell‐related osteogenesis in PDLs, contributing to the maintenance of periodontal homeostasis.^[^ ^57^ ^]^ Therefore, it is important to investigate whether viscoelastic hydrogels can benefit PDL healing in vitro under external stress loads. Compared with viscoelastic alginate hydrogels or collagen hydrogels,^[^ ^21^, ^43^, ^54^ ^]^ the physiochemical properties of F127DA hydrogels remain stable during investigation of the effects of viscoelastic properties on cell behavior, as demonstrated by the fact that they undergo little degradation in PBS or by cells.^[^ ^58^ ^]^ Interestingly, our data showed that PDLSCs cultured on F127DA‐5 hydrogels showed maximal fibrogenic differentiation potential under cytomechanical loading, while no significant differences could be observed when cells were cultured on F127DA hydrogels under static conditions (Figure 3). Although both viscoelasticity and the elastic modulus could influence the cellular behavior of PDLSCs, the forces that cells exert on the elastic matrix remained constant over time.^[^ ^46^ ^]^ The viscoelastic matrix can relax contraction forces gradually, leading to increased cell spreading and ligand clustering.^[^ ^21^, ^43^ ^]^ Considering that cells can bind to the ECM within seconds and form stable adhesion within minutes^[^ ^59^, ^60^ ^]^ and that external mechanical stress can reinforce such mechanotransduction,^[^ ^46^, ^61^ ^]^ the fast stress relaxation of the ECM is critical for actin polymerization and stress fiber formation in cells, which in turn contributes to robust cytoskeletal organization. The slow stress relaxation of F127DA‐10 or F127DA‐20 hydrogels might inhibit cell remodeling in ECMs, thereby inhibiting the related cellular mechanotransduction and fibrogenic differentiation of PDLSCs. Together, these data indicated that mechanical stress can change the bioactive effects of materials, and the fast stress relaxation of F127DA hydrogels, but not the elastic modulus, could harness mechanical loads to promote the fibrogenic differentiation of PDLSCs.

### 2.4 The Viscoelastic F127DA Hydrogels Influence the Fibrogenic Differentiation Potential of PDLSCs by Regulating Cell–ECM Interactions under Cytomechanical Loading

**Figure 4:**
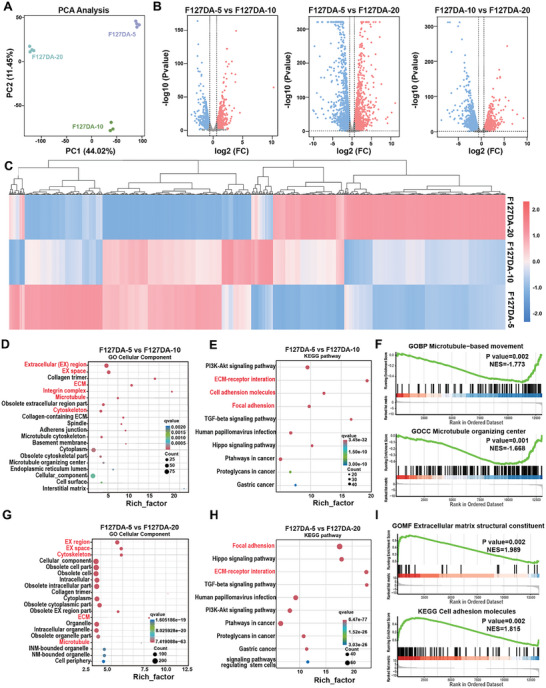
RNA‐seq revealed that viscoelastic F127DA hydrogels influenced the cell–ECM interaction under cytomechanical loading. A) PCA of genes from PDLSCs cultured on F127DA‐5, F127DA‐10 hydrogels, and F127DA‐20 hydrogels. B) Volcano plots of the differentially expressed genes (fold change >1.5 and adjusted *p* < 0.05) in PDLSCs cultured on F127DA‐5 versus F127DA‐10 hydrogels, F127DA‐5 versus F127DA‐20 hydrogels, and F127DA‐10 versus F127DA‐20 hydrogels. C) Heat map of RNA‐seq data showing differently expressed genes among PDLSCs cultured on F127DA‐5, F127DA‐10, and F127DA‐20 hydrogels after cluster analysis (*n* = 3–4). D) GO enrichment analysis of differently expressed genes in PDLSCs cultured on F127DA‐5 and F127DA‐10 hydrogels. E) Top KEGG enrichment analysis of potential pathways targets for fibrogenic differentiation of PDLSCs cultured on F127DA‐5 and F127DA‐10 hydrogels. F) GSEA analysis of pathways (GOBP microtubule‐based movement and GOCC microtubule organizing center) involved in cytoskeleton rearrangement of PDLSCs cultured on F127DA‐5 and F127DA‐10 hydrogels. G) GO enrichment analysis of differently expressed genes in PDLSCs cultured on F127DA‐5 and F127DA‐20 hydrogels. H) Top KEGG enrichment analysis of potential pathways targets for fibrogenic differentiation of PDLSCs cultured on F127DA‐5 and F127DA‐20 hydrogels. I) GSEA analysis of pathways (GOMF extracellular matrix structural constituent and KEGG Cell adhesion molecules) involved in cell–ECM interactions of PDLSCs cuktured on F127DA‐5 and F127DA‐20 hydrogels. Three to four samples were measured per group.

To gain insight into the underlying mechanism of viscoelastic F127DA hydrogels on fibrogenic differentiation of PDLSCs, the differently expressed genes in PDLSCs cultured on F127DA‐5, F127DA‐10, and F127DA‐20 hydrogels were analyzed by a bulk RNA sequencing (RNA‐seq). Under static conditions, the principal component analysis (PCA) showed the diffused distribution of duplicated plots, and no obvious clusters could be observed, indicating similar gene expression profiles among different groups (Figure S3, Supporting Information). Consistently, there were a few differentially expressed genes among different groups as shown by the volcano plots and heat maps (Figures S4–S6, Supporting Information). Under cytomechanical loadings, the PCA revealed clustered plots in PDLSCs cultured on F127DA‐5, F127DA‐10, and F127DA‐20 hydrogels, with PC1 and PC2 values of 44.02% and 11.45%, respectively, indicating the significant different gene expression profiles among the cohorts (**Figure** **4**A). As shown by the volcano plots, there were 799 significantly upregulated genes and 614 significantly downregulated genes between F127DA‐5 and F127DA‐10 groups, 2102 significantly upregulated genes, and 2231 significantly downregulated genes between F127DA‐5 and F127DA‐20 groups, and 1390 significantly upregulated genes and 1781 significantly downregulated genes between F127DA‐10 and F127DA‐20 groups (Figure 4B). The differently expressed genes were plotted as a heatmap in Figure 4C, indicating the different influences elicited by viscoelastic F127DA‐5, F127DA‐10, and F127DA‐20 hydrogels, respectively. The biological functions and process of differently expressed genes were further predicted and analyzed by Gene ontology (GO) cellular component enrichment analysis. The top 9 enriched biological processes in PDLSCs cultured on F127DA‐5 and F127DA‐10 hydrogels were involved in the ECM remodeling, integrin complex, and cytoskeleton rearrangement (Figure 4D). Consistently, the Kyoto Encyclopedia of Genes and Genomes (KEGG) enrichment analysis also indicated that most differently expressed genes were involved in cell–ECM interactions, such as ECM‐receptor interaction, cell adhesion molecules, and focal adhesion (Figure 4E). Gene set enrichment analysis (GSEA) also showed that the differently expressed genes between F127DA‐5 and F127DA‐10 groups are associated with the cytoskeleton rearrangement of PDLSCs (gene ontology biological processes (GOBP) Microtubule‐based movement and gene ontology cellular component (GOCC) Microtubule organizing center) (Figure 4F). In line with these results, further analysis via GO cellular component and KEGG enrichment analysis showed that the differently expressed genes between F127DA‐5 and F127DA‐20 groups, and F127DA‐10 and F127DA‐20 groups participated in the ECM remodel and cytoskeleton rearrangement, and the enriched signaling pathways highlighted focal adhesion and ECM‐receptor interaction (Figure 4G,H and Figure S7, Supporting Information). GSEA analysis also revealed that the differently expressed genes were associated with the extracellular organization and cell adhesion (Figure 4I). Overall, these findings indicated that viscoelastic F127DA hydrogels could harness the mechanical stress to enhance cell–ECM interactions, thereby influencing the fibrogenic differentiation of PDLSCs.

### 2.5 Viscoelastic F127DA Hydrogels Enhanced Integrin–FAK Signaling and the Cytoskeletal Rearrangement of PDLSCs under Cytomechanical Loading

**Figure 5:**
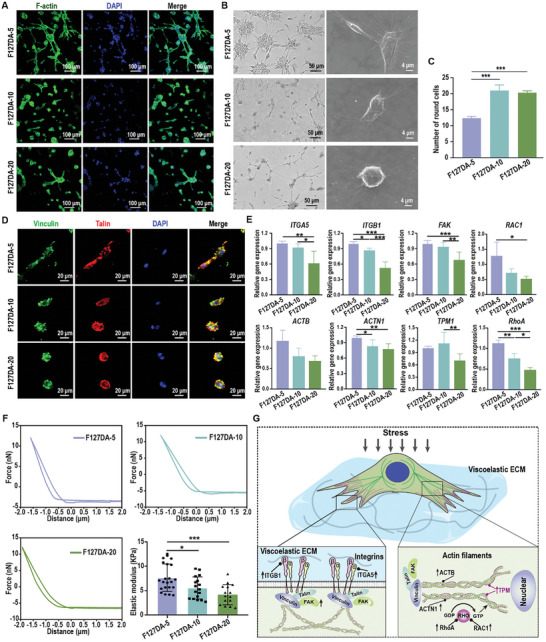
The mechanical responses of PDLSCs toward viscoelastic F127DA hydrogels under cytomechanical loading (≈0–120 KPa, 1 Hz, 1 h day^−1^) for 24 h. A) Representative images of phalloidin staining showing the cytoskeleton of PDLSCs cultured on F127DA‐5, sF127DA‐10, and F127DA‐20 hydrogels under cytomechanical loading. Scale bar = 100 µm. B) Representative optical microscopy (left panels) and SEM (right panels) images showing the morphology of PDLSCs cultured on F127DA‐5, F127DA‐10, and F127DA‐20 hydrogels under cytomechanical loading. Scale bar = 50 µm (left panels) or 4 µm (right panels). C) Quantitative analysis of nonspread round cells cultured on F127DA‐5, F127DA‐10, and F127DA‐20 hydrogels under cytomechanical loading (*n* = 3). D) Representative images of immunofluorescence staining showing the focal adhesion related markers (vinculin and talin) of PDLSCs cultured on F127DA‐5, F127DA‐10, and F127DA‐20 hydrogels under cytomechanical loading. Scale bar = 20 µm. E) The expression levels of integrin–FAK pathway‐related genes (*ITGA5, ITGB1, FAK* and *RAC1)* and cytoskeletal rearrangement‐related genes (*ACTINB*, *ACTIN1, TPM1* and *RhoA*) in PDLSCs cultured on F127DA‐5, F127DA‐10 and F127DA‐20 hydrogels under cytomechanical loading (*n* = 3–6) (qRT–PCR assay). F) Representative deflection–distance curves and calculated elastic modulus of PDLSCs analyzed by AFM under cytomechanical loading (*n* = 15–22). G) Schematic diagram showing the mechanical regulation of viscoelastic hydrogels on integrin–FAK pathways and cytoskeletal rearrangement of PDLSCs under cytomechanical loading. The data are shown as the mean ± SD; Data shown in (C), (E), and (F) were analyzed by one‐way ANOVA; *p < 0.05, **p < 0.05, and ***p < 0.001 indicate significant differences between the indicated columns.

The activation of integrin–FAK signaling plays a key role in regulating cell–ECM interactions by linking the ECM to intracellular signaling.^[^ ^62^, ^63^ ^]^ Upon binding to the ECM, integrin clusters form focal adhesion or multiprotein hubs that connect to the cytoskeleton.^[^ ^64^, ^65^ ^]^ The physical properties of the ECM, including viscoelasticity and elastic modulus, can influence integrin clusters to activate integrin–FAK signaling and cytoskeletal rearrangements, including actin bundling and stress fiber formation, thereby influencing cell spreading and intracellular signaling pathways.^[^ ^54^, ^66^ ^]^ Combined with the physical properties of the ECM, external mechanical loads can also trigger integrin–FAK signaling to generate tension and determine cell fates.^[^ ^67^ ^]^ Therefore, we hypothesized that integrin–FAK signaling and cytoskeletal rearrangement were involved in F127DA‐5 hydrogel‐mediated cell–ECM interactions under cytomechanical loads. After 24 h of cytomechanical loading, the spreading and morphology of PDLSCs were evaluated by phalloidin staining, optical microscopy, and SEM. Representative images of phalloidin‐stained cells showed that PDLSCs cultured on viscoelastic F127DA‐5 hydrogels displayed a larger spreading morphology than those cultured on F127DA‐10 and F127DA‐20 hydrogels (**Figure** **5**A). Consistent with phalloidin staining, representative optical microscopy, and SEM images both showed that PDLSCs cultured on F127DA‐5 hydrogels with faster stress relaxation rates were spindle‐shaped, and many more filopodia or lamellipodia could be observed. In contrast, PDLSCs cultured on F127DA‐20 hydrogels were round or oval, and only a few filopodia or lamellipodia could be observed (Figure 5B). Consistently, the quantification also showed that the number of nonspreading round cells in the F127DA‐5 group was much lower than that in the F127DA‐10 or F127DA‐20 group (Figure 5C). The immunofluorescent staining revealed more vinclin and talin expression in cells cultured on F127DA‐5 hydrogels, indicating more formation of focal adhesions (Figure 5D). Furthermore, qRT–PCR analysis revealed that PDLSCs cultured on viscoelastic F127DA‐5 hydrogels exhibited higher expression levels of integrin–FAK pathway‐related genes (*Integrin* *Alpha* (*ITGA)5, Integrin Beta (ITGB1), FAK*, and *Ras‐related C3 botulinum toxin substrate 1* *(RAC1))* than cells cultured on the F127DA‐10 and F127DA‐20 hydrogels under cytomechanical loading (Figure 5E). *ITGA‐5* and *ITGB1* encode integrin α5β1 and integrin β1, which are major components of integrins. *FAK* encodes FAK. Integrin α5β1 and integrin β1 switch between relaxed and tensioned states, which is necessary for external mechanical load‐induced FAK activation.^[^ ^67^, ^68^, ^69^ ^]^ The spatiotemporal RAC1 can influence the stability of integrin adhesions.^[^ ^62^ ^]^ Under cytomechanical loads, F127DA‐5 hydrogels with fast stress relaxation allowed the rapid remodeling of ECM to promote the clustering of integrin α5β1 and integrin β1, thereby activating integrin–FAK signaling. In contrast, the low stress relaxation of F127DA‐10 or F127DA‐20 hydrogels compromised the spreading and activation of integrins, which might be why the spreading of PDLSCs was still inhibited under cytomechanical loads, and these inhibitory influences cannot be reversed by the high elastic modulus. Following the activation of integrin–FAK signaling, the polymerization/depolymerization of the actin cytoskeleton mediates the cytoplasmic‐to‐nuclear localization of various transcription factors.^[^ ^70^ ^]^ qRT‒PCR analysis showed that cytomechanical loads caused a greater increase in the expression of cytoskeletal rearrangement‐related genes (*Actin Beta* *(ACTB), Actin‐1 (ACTN1)* and *Tropomyosin‐1* (*TPM1)*) in cells cultured on F127DA‐5 hydrogels than in those cultured on F127DA‐10 and F127DA‐20 hydrogels(Figure 5F). Meanwhile, Ras Homolog Family Member A (*RhoA)* also increased significantly in F127DA‐5 groups, indicating the active actin polymerization.^[^ ^62^, ^70^ ^]^ The activation of integrins and polymerization of actins increased the force on the resistant plasma membrane, consequently increasing the cellular traction force on the ECM (Figure 5F). Together, these findings suggest that under cytomechanical loading, F127DA‐5 hydrogels with faster stress relaxation rates can promote integrin α_5_β_1_ and FAK activation and cytoskeletal rearrangement in PDLSCs (Figure 5G).

**Figure 6:**
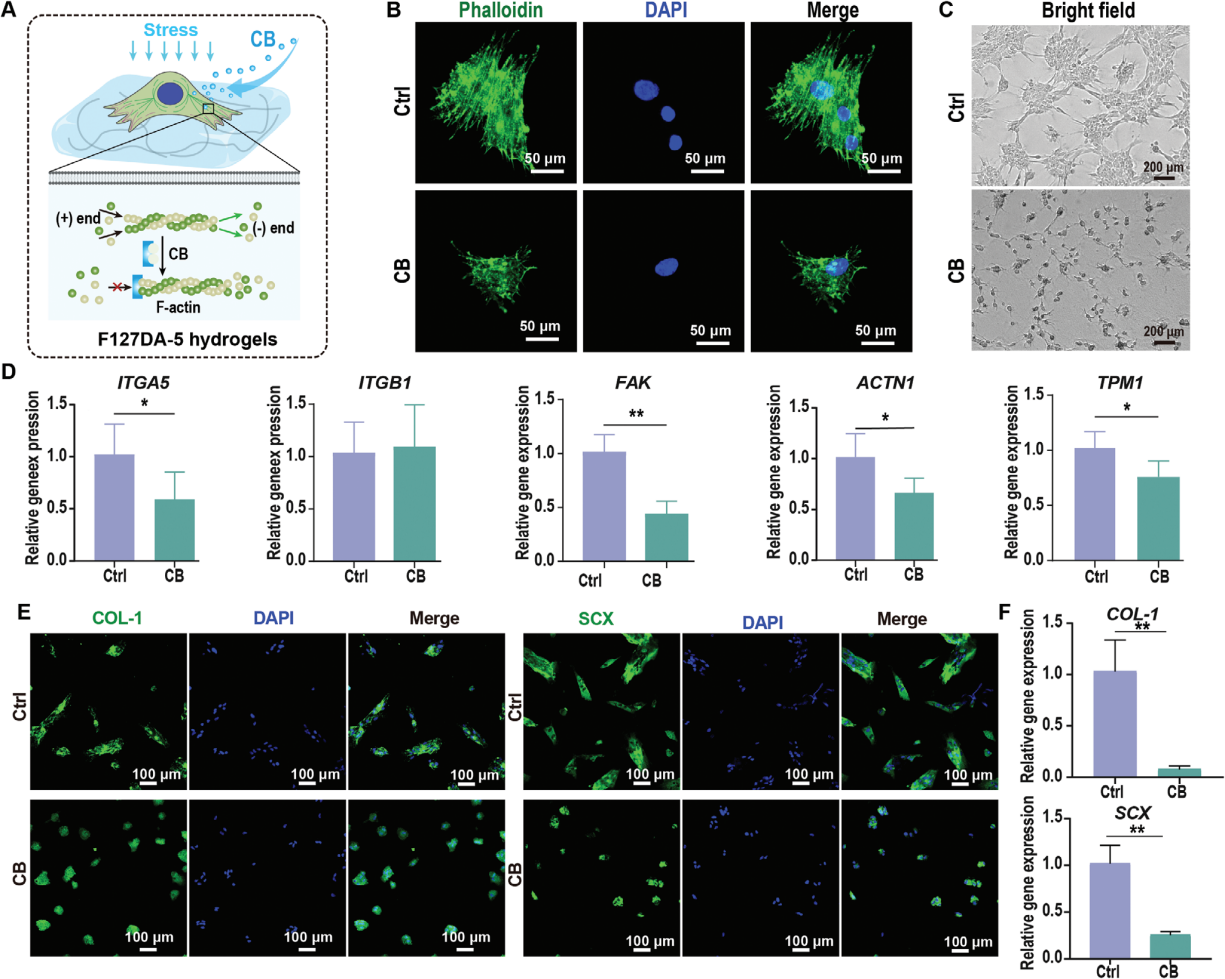
The effects of CB on the mechanical responses and fibrogenic differentiation of PDLSCs cultured on viscoelastic F127DA‐5 hydrogels under cytomechanical loading (≈0–120 KPa, 1 Hz, 1 h day^−1^). A) Schematic diagrams showing that the actin polymerization of PDLSCs cultured on viscoelastic F127DA‐5 hydrogels was inhibited by 10 µm CB under cytomechanical loading. B) Representative images of phalloidin staining showing the influence of CB treatment on the cytoskeletons of PDLSCs grown on viscoelastic F127DA‐5 hydrogel under cytomechanical loading. Scale bar = 50 µm. C) Representative optical microscopy images showing the influence of CB treatment on the viscoelastic F127DA‐5 hydrogel‐mediated morphology of PDLSCs. Scale bar = 200 µm. D) Decreased expression levels of integrin–FAK pathway‐related genes (*ITGA5, ITGB1, FAK*) and cytoskeletal rearrangement‐related genes (*ACTN1* and *TPM1*) in PDLSCs following CB incubation under cytomechanical loading (*n* = 6) (qRT–PCR assay). E) Representative images of immunofluorescence staining showing the effects of CB treatment on the expression of fibrogenic differentiation‐related markers (COL‐1 and SCX) in PDLSCs under cytomechanical loading. Scale bar = 100 µm. F) Decreased expression levels of fibrogenic differentiation‐related genes (*COL‐1* and *SCX*) in PDLSCs following CB incubation under cytomechanical loading (*n* = 3) (qRT–PCR assay). The data are shown as the mean ± SD. Data shown in (D) and (F) were analyzed by unpaired *t* test; *p < 0.05 and **p < 0.001 indicate significant differences between the indicated columns.

To further study the effect of cytoskeletal rearrangement on the fibrogenic differentiation potential of PDLSCs, cytochalasin B (CB) was used to inhibit the actin cytoskeleton of PDLSCs cultured on F127DA‐5 hydrogels under cytomechanical loading. Representative phalloidin staining images after treatment with 10 µM CB for 24 h showed that CB treatment resulted in shortened actin fibers forming cytoplasmic puncta under cytomechanical loading (**Figure** **6**A,B). Consistent with the phalloidin staining results, representative optical microscopy images showed that cells cultured on F127DA‐5 hydrogels became round after CB treatment (Figure 6C). To confirm the effects of cytoskeletal inhibition on the ECM–integrin–cytoskeleton linkage of PDLSCs on F127DA‐5 hydrogels, qRT–PCR analysis revealed that CB treatment significantly decreased the expression levels of integrin–FAK pathway‐related genes (*ITGA5, ITGB1, FAK)* and cytoskeletal rearrangement‐related genes (*ACTN1* and *TPM1*) (Figure 6D). The effect of cytoskeletal inhibition on the fibrogenic differentiation potential of PDLSCs under cytomechanical loading was further studied. After treatment with 10 µM CB for 7 days under cytomechanical loading, representative immunofluorescence staining images revealed that the presence of fibrogenic differentiation‐related markers (COL‐1 and SCX) in PDLSCs cultured on the F127DA‐5 hydrogels with CB treatment was less than that in the control (Figure 6E). Consistent with the immunofluorescence staining results, qRT–PCR analysis revealed that PDLSCs cultured on viscoelastic F127DA‐5 hydrogels with CB treatment exhibited lower expression levels of fibrogenic differentiation‐related genes (*COL‐1* and *SCX*) than control PDLSCs (Figure 6F). Taken together, our results indicate that cytoskeletal rearrangement plays a key role in the viscoelastic F127DA‐5‐enhanced fibrogenic differentiation potential of PDLSCs under mechanical loading and that inhibiting the formation of actin microfilaments by CB could abolish the mechanical response of viscoelastic F127DA‐5 hydrogels.

Notably, the elastic modulus and viscoelasticity of F127DA hydrogels were both changed in the present study, making the data presentation and elucidation chaotic. In the future, viscoelastic hydrogels with tunable stress relaxation independent of a high elastic modulus should be designed. Further studies can also focus on the involvement of ECM–integrin–cytoskeleton linkages in viscoelastic hydrogel‐enhanced fibrogenic differentiation under cytomechanical loading.

### 2.6 Viscoelastic F127DA Hydrogels Promoted the Repair and Regeneration of PDLs in the Delayed Replantation of Avulsed Teeth

**Figure 7:**
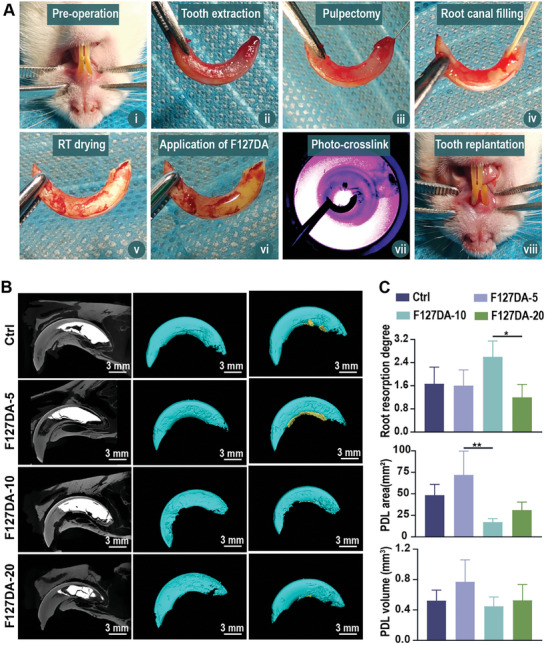
The effects of viscoelastic F127DA hydrogels on tooth resorption and PDL healing in the delayed replantation of avulsed teeth in rats. A) Representative photographs showing the delayed replantation of avulsed incisors in rat. i) Preoperative photographs of maxillary incisors in rat. ii) Extraction of the maxillary left incisor in rat. iii) Removal of the dental papilla and pulp tissues. iv) Filling of root canal with calcium hydroxide. v) Extracted maxillary incisor after air‐dried in a fume hood for 60 min. vi) Application of F127DA hydrogels on the root surface. vii) Photo‐crosslinking of F127DA hydrogels. viii) Replantation of the F127DA hydrogel‐coated maxillary incisor. B) Representative images of micro‐CT scanning showing tooth resorption and newly formed PDLs alongside the tooth root at 8 weeks after delayed tooth replantation. Left panels: representative 2D micro‐CT images showing the PDL space between the tooth root and lamina dura. Middle panel: 3D reconstruction of micro‐CT images showing the morphology of the tooth root surface (cyan). Right panel: 3D reconstruction of micro‐CT images showing the newly formed PDLs surrounding the tooth root (yellow). Scale bar = 3 mm. C) Quantitative analysis of root resorption degree, PDL area, and PDL volume at 8 weeks post surgery based on micro‐CT scanning (*n* = 5). The data are shown as the mean ± SD. Data shown in (C) were analyzed by Kruskal‒Wallis test; *p < 0.05 and **p < 0.01 indicate significant differences between the indicated columns.

Due to the hierarchical structure and complex mechanical microenvironments, restoring lost PDL attachments in mechanically stimulated environments continues to be a crucial yet challenging task in the field of periodontal tissue engineering.^[^ ^11^, ^71^ ^]^ Considering that the viscoelasticity of PDLs can structurally modulate the mechanical response of the tooth–PDL–bone complex and facilitate the remodeling of the periodontium under mechanical stimulation,^[^ ^72^, ^73^, ^74^ ^]^ we hypothesized that viscoelastic hydrogels could promote PDL regeneration and healing under occlusal stimulation. First, the biodegradation process of F127DA hydrogels was monitored following subcutaneous implantation. The representative images of in vivo fluorescence demonstrated that F127DA‐5, F127DA‐10, and F127DA‐20 hydrogels could be degraded over time, but they all remained in the implantation sites for 2 weeks (Figure S8A,B, Supporting Information). These results indicated that F127DA hydrogels could retain in *vivo* and provide stable mechanical supports for weeks to allow the regeneration and remodeling of PDLs. As a proof of concept, the delayed replantation of an avulsed incisor model in rats was established to evaluate the in vivo therapeutic performance of viscoelastic F127DA hydrogels on PDLs. One of the advantages of the delayed replantation of the avulsed tooth model is that PDLs can be completely necrotized by air drying for 1 h, while damage to alveolar bones can be minimized.^[^ ^75^ ^]^ The other advantage is that the occlusal force can be controlled by altering the food structure (feeding with jelly or solid feed).^[^ ^76^ ^]^ To establish the delayed replantation of the avulsed tooth model, the maxillary left incisor was first extracted from the tooth fossa, and then the extracted incisor was air‐dried in a fume hood for 60 min to ensure complete necrosis of the PDLs. Subsequently, F127DA‐5, F127DA‐10, or F127DA‐20 hydrogel was coated on the surface of roots. After photo‐crosslinking, the hydrogel‐coated tooth was replanted into the corresponding extraction socket (**Figure** **7**A). During the experiment, all the animals exhibited good wound healing at the replantation sites, and no severe adverse events occurred, so all animals were used for the subsequent tests. At 8 weeks post surgery, all the animals were euthanized, and the maxillaries were harvested to investigate the therapeutic effects of viscoelastic F127DA hydrogels on PDL injury by micron‐scale computed tomography (micro‐CT) and H&E, Masson, and immunofluorescence staining. As shown in Figure 7B, representative micro‐CT scanning images showed that tooth resorption was present in all the tested groups. However, the resorption sites were more easily visualized on the surface of replanted teeth coated with F127DA‐10 hydrogels. The absence or enlargement of PDL space could also be observed in the Control or F127DA‐10 groups. Consistently, the three‐dimensionally reconstructed images showed large amounts of resorption pits on the replanted root surfaces (green), and newly formed PDLs (yellow) could be observed covering the root surface. Compared with the F127DA‐10 and F127DA‐20 groups, more PDLs formed alongside the root surface in the F127DA‐5 group (Figure 7B). As demonstrated by quantitative analysis, the degree of root resorption in the F127DA‐10 group was the highest among all tested groups. The area and volume of newly formed PDLs in the F127DA‐5 group were the highest, but there was no significant difference in PDL volume among the four groups (Figure 7C).

**Figure 8:**
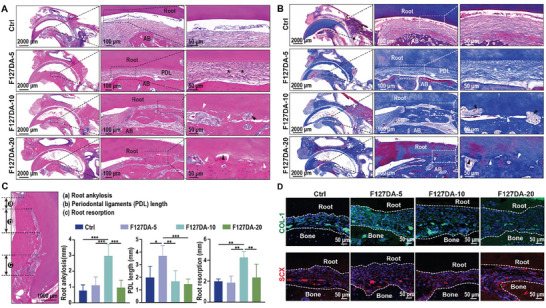
Therapeutic performance of viscoelastic F127DA hydrogels on PDL injury in delayed replantation of avulsed teeth. Tooth roots that did not receive any implants were used as Ctrl. Representative images of A) H&E staining and B) Masson staining showing the newly formed PDLs, root resorption, and ankylosis at 8 weeks after the delayed replantation of avulsed incisors. Left panels: overview of the teeth replanted in the alveolar sockets. Scale bar = 2000 µm); middle and right panels: magnified view (marked region on the left) showing the PDLs (asterisks), inflammatory or replacement tooth resorption (black triangles), or ankylosis (white triangles). Scale bar = 100 µm (middle panels) or 50 µm (right panels). C) Histometric analysis of root ankylosis, PDL length, and root resorption in tissue slices from each group (*n* = 6). Landmarks and parameters used for histometric analysis are shown in the representative H&E staining image (left). D) Representative images of immunofluorescence staining showing the expression of COL‐1 and SCX across the newly formed PDL tissues (the dotted lines indicate the bone–ligament or ligament–root interfaces). The data are shown as the mean ± SD. Data shown in (C) were analyzed by one‐way ANOVA; *p < 0.05, **p < 0.005, and ***p < 0.001 indicate significant differences between the indicated columns.

To further evaluate the regenerative outcomes of PDLs, tooth sections representing the whole length of replanted incisors were prepared and then subjected to H&E and Masson staining. Representative images of H&E and Masson staining showed that the interstitial space between alveolar bone and the replanted root was filled with newly formed PDL‐like connective tissue in all groups. Specifically, the PDL‐like tissues in the F127DA‐5 groups consisted of dense collagen fibers, and the newly formed collagen fibers were inserted perpendicularly into the cementum on the root surface, similar to normal PDLs. However, in the F127DA‐10 and F127DA‐20 groups, inflammation, replacement tooth resorption, and ankylosis were frequently observed (**Figure** **8**A,B). Quantitative histological analysis demonstrated that the PDL length in the F127DA‐5 group was the greatest among all tested groups. In contrast, the lengths of tooth resorption and root ankylosis in the F127DA‐10 group were the highest (Figure 8C). The potential cause for F127DA‐10‐induced root resorption may be attributed to the fact that medium stiffness (≈44.6 kPa) facilitated the greatest formation of preosteoclasts, reinforcing the stiff matrix‐mediated osteoclastic differentiation when the elastic modulus of matrix exceeds 29.4 Kpa.^[^ ^77^ ^]^ Consistent with the histological analysis, representative immunofluorescence images showed that COL‐1 and SCX (fibrogenic differentiation‐related markers) were highly expressed in newly formed PDL‐like tissues (Figure 8D). These findings were in line with previously published research in which viscoelastic alginate‐based hydrogels also promoted PDL regeneration. However, both bone and PDL were removed in that study,^[^ ^12^ ^]^ which disturbed the mechanical pathways from tooth to alveolar bone. Therefore, the present study provided more substantial evidence that viscoelastic biomaterials could promote PDL regeneration under mechanical stimulation. However, it is still unclear whether viscoelastic biomaterials matching the fast stress relaxation of PDLs can restore the mechanical microenvironments of PDLs due to the challenges in measuring the mechanical stress of PDLs in vivo. Advances in biomechanical testing will help to identify and select parameters for designing PDL‐mimicking biomaterials, which can reshape the mechanical adaptability and promote the tissue regeneration of PDLs. For clinical applications of F127DA hydrogels, the therapeutic effects of F127DA hydrogels on PDL regeneration should be further analyzed on large animals, such as nonhuman primates, dogs, sheeps, or mini pigs. Moreover, further optimization is required for the adhesion and binding of F127DA hydrogels to the adjacent cementum and alveolar bones.

## 3 Conclusions

Mechanical stress can act as a biological stressor that elicits a homeostasis response to reshape mechanical adaptability and promote the reconstruction of injured PDLs. However, mechanical homeostasis is easily lost but difficult to restore. Inspired by the excellent viscoelasticity of PDLs, which can withstand and disperse chewing or biting forces, we first demonstrated an approach to modulate the viscoelasticity and elastic modulus of F127DA hydrogels, which could match the fast relaxation rates of PDLs and provide mechanical support without chemical cross‐linking. Then, we found that the F127DA hydrogels with the fastest stress relaxation harnessed cytomechanical loads to improve the fibrogenic differentiation potential of PDLSCs. Mechanically, the viscoelastic F127DA hydrogels could activate the mechanotransduction of PDLSCs by upregulating integrin–FAK pathways and related cytoskeletal rearrangement under cytomechanical loading, leading to reinforced cell–ECM interactions. The in vivo experiments also indicated that the F127DA hydrogels with the fastest stress relaxation significantly promoted the repair of PDLs and reduced abnormal healing (e.g., root resorption and ankyloses) in the delayed replantation of avulsed teeth. These findings provide a novel strategy to promote the regeneration of PDLs and elucidate the important roles of mechanical stress in efforts to develop functional PDL regeneration.

## 4 Experimental Section

### Synthesis of Pluronic F127DA Hydrogels

F127DA hydrogels were synthesized according to previously published studies.^[^ ^78^, ^79^ ^]^ Briefly, 10 g of F127 (Sigma Aldrich, St. Louis, MO, USA) was dissolved in 100 mL of anhydrous dichloromethane (Aladdin, Shanghai, China) with constant stirring. After complete dissolution, triethylamine (ACMEC biochemical, Shanghai, China) and acryloyl chloride (Aladdin) were slowly added. The mixture was reacted in an ice bath for 1 h and then kept at room temperature for 24 h. Subsequently, the precipitated triethylamine was removed by infiltration, and the residual filtrate was precipitated slowly by adding supercooled anhydrous diethyl ether. Then, F127DA was obtained by drying in vacuum at 30 °C for 24 h. For the generation of F127DA hydrogels, F127DA was dissolved in 0.5% (wt/vol) lithium acylphosphinate photoinitiator (LAP; EngineeringForLife, Suzhou, Jiangsu, China) at various concentrations (wt/vol; 5%, 10% and 20%) at 4 °C. After incubation for 1 h, the hydrogel solutions containing different concentrations of F127DA were simultaneously solidified through exposure to 30 mW cm^−2^ 405 nm UV light for 30 s using a 405 nm light‐curing portable source (EFL, EFL‐LS‐1601‐405, China). Final gelatin containing 5%, 10%, or 20% F127DA in the synthesized hydrogels was referred to as F127DA‐5, F127DA‐10, or F127DA‐20 hydrogels, respectively.

### FT‐IR Analysis of F127 and F127DA Hydrogels

The FT‐IR spectra of F127 and F127DA were recorded at resolutions of 400–4000 and 4 cm^−1^ by an FT‐IR spectrometer (Thermo Scientific Nicolet iS5, ThermoFisher Scientific, Waltham, MA, USA).

### Microstructure of F127DA Hydrogels

The microstructure of the synthesized F127DA‐5, F127DA‐10, or F127DA‐20 hydrogels was first characterized by SEM (Hitachi S‐4800, EIKO Engineering, Tokyo, Japan). The synthesized F127DA was dissolved in 0.5% (wt/vol) LAP and injected into a cylindrical mold. After UV curing, the cylindrical F127DA hydrogels were precooled at −20 °C for 4–5 h and −80 °C for 18 h and then freeze‐dried using a vacuum‐freeze drying device (GOLD‐SIM, LA, California, USA). To observe the inner microstructure of the F127DA hydrogels, the lyophilized hydrogels were cut into slices of 1 mm thickness. Then, the fracture morphologies were observed and imaged by SEM (Hitachi S‐4800).

### The Porous Properties of F127DA Hydrogels

The liquid displacement method was performed to determine the porosity of F127DA‐5, F127DA‐10, and F127DA‐20 hydrogels, respectively. 100 µL of F127DA solutions were added into 1‐mL syringes to fabricate regular cylindrical F127DA hydrogel samples. After freeze‐drying, the F127DA‐5, F127DA‐10, and F127DA‐20 hydrogels were weighed for their dry weight (*W*_d_). Subsequently, they were immersed in ethanol and evacuated under vacuum until no air bubbles were present. After 1 h, the hydrogels were suspended in the ethanol, and the weight of F127DA‐5, F127DA‐10, and F127DA‐20 hydrogels immersed in the ethanol (*W*_e_) was determined. The hydrogels were then taken out from the ethanol, the excessive ethanol adsorbed on the hydrogel was gently removed, and the weight of F127DA‐5, F127DA‐10, and F127DA‐20 hydrogels in air (*W*_a_) were measured. The porosity (*P* (%)) is defined as,

(1) $$P\left(\%\right)=\frac{V_{p}}{V_{s}}=\frac{\left(W_{a}-W_{d}\right)/\rho_{\mathrm{eth}}}{\left(W_{a}-W_{e}\right)/\rho_{\mathrm{eth}}}=\frac{W_{a}-W_{d}}{W_{a}-W_{e}}$$

where *V*_p_ is the pore volume, *V*_s_ is the total volume of pores and solid matrix, *ρ*_eth_ is the density of ethanol, *W*_e_ is the wet weight of the hydrogels immersed in ethanol, *W*_d_ is the dry weight of the hydrogels, and *W_a_* is the weight of the hydrogels in air after ethanol immersion.

### The Mechanical Properties of F127DA Hydrogels

The compression modulus of the F127DA hydrogel was measured by a biomechanical tester (EFL‐MT‐5600, EngineeringForLife) according to previously published methods.^[^ ^80^ ^]^ Briefly, hydrogel samples were cast in a 2‐mL syringe (6.5 mm in height). Then, the compression test was performed with a drop speed of 1 mm min^−1^ until the pressure reached a maximum of 20 N in the loading period. In the unloading period, the plate rise speed was set to 1 mm min^−1^ until the pressure dropped to a minimum of 0.001 N. The elastic modulus of each sample was calculated as the slope of the resulting loading curves in the linear regions (first 0 to 10% of strain) generated by the corresponding software (EFL‐MT‐5600, EngineeringForLife). The area enclosed by the loading–unloading curve was defined as the energy dissipated by the hydrogel network. The energy dissipation was calculated using the formulas below.

(2) $$\Delta U=\underset{0}{\int^{\varepsilon \left(\max-\mathrm{loading}\right)}}\sigma d\varepsilon -\underset{0}{\int^{\varepsilon \left(\max-\mathrm{un}\mathrm{loading}\right)}}\sigma d\varepsilon$$

where Δ*U* is the dissipation energy (J m^−3^), *σ* denotes stress (Pa), *ε* represents strain (%), *ε*(max‐loading) is the maximum strain during loading period, and *ε*(max‐unloading) is the Maximum strain during unloading period.

To assess the mechanical stability of F127DA‐5, F127DA‐10, and F127DA‐20 hydrogels, the loading–unloading curves of F127DA‐5, F127DA‐10, and F127DA‐20 hydrogels were recorded for 5 cycles of compression tests using consistent methods.

### Rheological Characterization of F127DA Hydrogels

Rheological tests were carried out with a rotational rheometer equipped with a parallel plate geometry (plate diameter: 25 mm) (Anton Paar MCR 302, Anton Paar, Graz, Austria) according to the previously published literature.^[^ ^12^, ^43^ ^]^ Briefly, the F127DA‐5, F127DA‐10, and F127DA‐20 hydrogels were uniformly photo‐crosslinked in a 50‐mL syringe (1 mm in thickness). Then, the disk‐shaped hydrogels were immersed in phosphate buffer solution (PBS) to prevent dehydration. Subsequently, the storage modulus (G′) and loss modulus (G″) of each sample were recorded using a frequency sweep test under 1% strain amplitude with a range of frequencies from 0.1 to 100 rad s^−1^. For the stress relaxation experiments, a constant strain of 1% was applied to each hydrogel at 37 °C, and the resulting shear stress was recorded over the course of 100 s. The half‐stress‐relaxation time (*τ*_1/2_) was calculated as the time at which the initial stress of the hydrogel was relaxed to half of its original value.

### The Isolation and Culture of hPDLSCs

hPDLSCs were isolated from permanent teeth without any dental or periodontal disease, provided by 6 systemically healthy donors (aged 18 to 30 years). All donors signed informed consent forms for the use of their extracted teeth in the research project, which was approved by the Ethics Committee of the School of Stomatology, Fourth Military Medical University, Xi'an, Shaanxi, China (grant no. IRB‐REV‐2022120). The isolation of PDLSCs was performed according to protocols reported in the previously published studies.^[^ ^30^, ^81^ ^]^ Briefly, PDL tissues were scraped from the middle third of the root surfaces. Then, the obtained PDL tissues were cut into pieces and digested in 3 mg mL^−1^ type I collagenase (DIYIBio, Shanghai, China) at 37 °C for 45 min. After digestion, the PDL tissues were incubated in α‐MEM (Gibco BRL, Grand Island, NY, USA) containing 10% fetal bovine serum (FBS; Gibco BRL) and 1% penicillin and streptomycin (Zimu Biology, Xi'an, Shaanxi, China), followed by transfer into 6‐well plates and culture in a humidified atmosphere with 5% CO_2_ at 37 °C. The medium was exchanged every 3 days. When primary cells migrated from PDL tissues and reached 80% confluence, the adherent cells were passaged with 0.25% trypsin (Zimu Biology). The PDLSCs at passages 3–5 were used in the subsequent experiments.

### The Adhesion and Morphology of PDLSCs Cultured on F127DA Hydrogels

The adhesion and morphology of PDLSCs cultured on F127DA‐5, F127DA‐10, and F127DA‐20 hydrogels were observed by optical microscopy, phalloidin staining, and SEM, respectively. Prior to cell cultivation, different concentrations of F127DA (wt/vol; 5%, 10%, and 20%) were dissolved in 0.5% (wt/vol) LAP at 4 °C. After complete dissolution, the F127DA hydrogel solutions were filtered through precooled 0.22‐µm filters. Subsequently, 120 µL of filtered hydrogel solutions with different concentrations (wt/vol; 5%, 10%, and 20%) were added to 48‐well plates per well and exposed to 30 mW cm^−2^ 405 nm UV light for 5 s to form initial hydrogels, followed by coating with 5 mg mL^−1^ RGDfK peptide acryloyl (EngineeringForLife) with UV light exposure for 25 s. All operations were carried out on ice to prevent automatic hydrogel gelation at room temperature. When the hydrogels were solidified, cells were seeded on the surfaces of F127DA‐5, F127DA‐10, or F127DA‐20 hydrogels in 48‐well plates at a density of 5 × 10^4^ cells per well. After 24 h of incubation, the number of nonspread round cells on the F127DA‐5, F127DA‐10, and F127DA‐20 hydrogels was observed by optical microscopy (Leica Microsystems, Heerbrugg, St. Gallen, Switzerland) and quantified according to previously published literature.^[^ ^82^ ^]^ For phalloidin staining, cells were rinsed three times with PBS and fixed in 4% paraformaldehyde (PFA; Servicebio, Wuhan, Hubei, China) for 15 min. After washing three times with PBS, cells were permeabilized with 0.1% Triton X‐100 (MP Biomedical, Irvine, California, USA) at room temperature for 5 min. Subsequently, the cells were rinsed and incubated in iFluor 488 phalloidin (1:500, 40736ES75, Yeasen, Shanghai, China) plus DAPI (1:1000, 40728ES03, Yeasen) for 1 h at room temperature in the dark. Representative images of phalloidin staining were captured by confocal microscopy (Nikon, Tokyo, Japan). For SEM examination, each sample was rinsed with PBS and fixed in 2.5% glutaraldehyde (Sigma Aldrich, St. Louis, MO, USA) for 6 h at room temperature. Then, the samples were dehydrated sequentially in an ethanol gradient (30, 50, 70, 80, 90, 100%) for 5 min. Subsequently, the hydrogels were placed in a fume hood to dry for 20 min. Finally, all samples were sputter‐coated with Pb/Au and imaged using field‐emission SEM (Hitachi S‐4800) according to the previous paper.^[^ ^83^ ^]^

### PDLSC Viability Cultured on F127DA Hydrogels

PDLSC viability cultured on F127DA hydrogels was characterized by live/dead cell staining and Cell counting Kit‐8 (CCK‐8) assays. For live/dead cell staining, 5 × 10^4^ cells were seeded on the surfaces of F127DA hydrogels as described before. After 1, 3, and 7 days of culture, the cells were rinsed with PBS and then incubated in 5 mL of PBS containing 5 µL of calcein‐AM (DIYIBio) plus 15 µL of propidium iodide (PI) (DIYIBio) for 15 min at room temperature in the dark. Representative images showing live/dead cells were then taken by confocal microscopy under the same setup parameters (Nikon), and randomly selected fields were used for image analysis using ImageJ 1.53k software. The cell viability was calculated as the number of living cells/the number of total cells (100%). For CCK‐8 assays, 2 × 10^3^ cells were seeded on the surfaces of F127DA hydrogels in 96 cell plates. After 1, 3, and 7 days of culture, each sample was rinsed with PBS and then incubated in 200 µL of serum‐free α‐MEM containing 20 µL of CCK‐8 reagent (DIYIBio) per well for 3 h at 37 °C in the dark. The absorbance at 450 nm was measured by a microplate reader (Tecan Infinite 200Pro, Tecan, Männedorf, Zurich, Switzerland).

### In Vivo Biocompatibility of F127DA Hydrogels

The biocompatibility of F127DA hydrogels was further investigated following subcutaneous implantation into the dorsal flanks of mice. Nine male C57 mice aged 7–8 weeks (purchased from the Laboratory Animal Center of FMMU) were used in the experiment. All surgical procedures were also approved by the Animal Research Committee of FMMU (grant no. IACUC‐20240113). The F127DA solutions were added into 2‐mL syringes to fabricate cylindrical F127DA hydrogel samples (3 mm in height). F127DA hydrogel samples were then subcutaneously transplanted into the bilateral backs of mice. At 2 weeks post surgery, the mice were euthanized. The full‐thickness skins covering the transplanted F127DA hydrogels and major organs of mice (liver, spleen, lung, heart, and kidney) were collected. The obtained samples were dehydrated, embedded in paraffin, sectioned with a thickness of 4 µm, and then stained with hematoxylin and eosin (H&E). The scanned slices were observed using slide‐viewing software (CaseViewer ver. 2.1, 3DHISTECH, Budapest, Pest megye, Hungary).

### The Elastic Modulus of PDLSCs Cultured on F127DA Hydrogels

1 × 10^5^ cells were seeded on the surfaces of F127DA‐5, F127DA‐10, or F127DA‐20 hydrogels (volume: 100 µL) in 35 mm glass bottom cell culture dishes. After 24 h of incubation, the cells were rinsed three times with PBS, and then the elastic modulus of PDLSCs was detected by AFM (Bruker, Billerica, Massachusetts, USA). The measurement was conducted in contact mode in an aqueous environment by using an AFM probe (PNP‐TR, Nanoworld, Neuchatel, Neuchâtel Canton, Switzerland) equipped with a triangular tip and with a nominal spring constant of 0.08 N m^−1^. The AFM tip was controlled to indent the cells in the approach–retract mode to measure the elastic properties of the cells, and the approach–retract curves were recorded by the AFM manipulation software. The elastic modulus of PDLSCs was calculated by the Sneddon model by using the following equation.

(3) $$F=\frac{2}{\pi }d^{2}\frac{E}{1-\nu^{2}}\tan\alpha$$

where *F* is the loading force of the AFM probe, *d* is the indentation depth, *E* is the Elastic modulus, *υ* is the Poisson ratio of cell (*υ* = 0.5), and *α* is the half‐opening angle of the conical tip.

### Fibrogenic Induction of PDLSCs on F127DA Hydrogels under Resting Conditions

PDLSCs were seeded on the surfaces of F127DA‐5, F127DA‐10, or F127DA‐20 hydrogels at a density of 5 × 10^4^ per well. When the cells reached 80–90% confluence, the culture medium was changed to fibrogenic induction medium, which was complete medium supplemented with 25 µg mL^−1^ corbic acid and 100 ng mL^−1^ connective tissue growth factor (CTGF; MedChemExpress, Monmouth Junction, NJ, USA) according to previously published literature.^[^ ^16^, ^84^ ^]^ The medium was changed every 3 days. After 7 days of incubation, the cells and supernatant were harvested for quantitative real‐time polymerase chain reaction (qRT‒PCR), immunofluorescence staining, and total collagen detection assays.

### The Fibrogenic Induction of PDLSCs on F127DA Hydrogels under Cytomechanical Loading

To mimic the hydrostatic pressure endured by cells encapsulated in the interstitial fluid‐filled ECM during mastication, a custom‐designed multifunctional in vitro cell compression system was used to exert dynamic compressive stress on PDLSCs according to the previously published studies.^[^ ^52^, ^53^ ^]^ The cells were first seeded on the surfaces of F127DA‐5, F127DA‐10 or F127DA‐20 hydrogels. When the cells reached 80–90% confluence, the culture medium was changed to fibrogenic induction medium. At 1, 3, and 5 days after fibrogenic induction, the cell–hydrogel constructs seeded in 48‐well plates were placed in the cell compression device and stimulated with pressure every other day. Based on previously published literature.^[^ ^52^, ^85^ ^]^ the pressure conditions were set to ≈0–120 kPa, 1 Hz, and 1 h day^−1^. After an incubation period of 7 days, the effects of viscoelastic F127DA hydrogels on the fibrogenic differentiation potential of PDLSCs under dynamic compressive stress were analyzed using qRT‒PCR, immunofluorescence staining and total collagen detection assays.

### qRT‒PCR

The expression levels of fibrogenic differentiation‐related genes (*SCX* and *COL‐1*) in PDLSCs cultured on viscoelastic F127DA hydrogels were analyzed by qRT‒PCR. Briefly, total cellular RNA was extracted and transcribed into complementary DNAs (cDNAs) using TRIzol Reagent (TIANGEN, Beijing, China) and a Hifair lll 1st Strand cDNA Synthesis Kit (Yeasen) according to the manufacturer's protocol. Then, qRT‒PCR was conducted using 2×qPCR SmArt Mix (SYBR Green) (No Rox) (DIYIBio). The primer sequences used in this study are listed in **Table** **1**.

**Table 1:** qRT–PCR primer sequences used in this study.

Gene	Full name	Gene ID	Primers	Sequences (5′‐3′)
*18S rRNA*	18S ribosomal RNA	106632259	Forward	CAGCCACCCGAGATTGAGCA
			Reverse	TAGTAGCGACGGGCGGTGTG
*COL‐1*	Collagen type I alpha 1 chain	1277	Forward	CTGACCTTCCTGCGCCTGATGTCC
			Reverse	GTCTGGGGCACCAACGTCCAAGGG
*SCX*	Scleraxis	642658	Forward	CCTGGCCTCCAGCTACATCT
			Reverse	TCGCGGTCCTTGCTCAACTTT
*ITGA5*	Integrin subunit alpha 5	3678	Forward	GAGGCAGTGCTATTCCCAGTAAG
			Reverse	GTCCCGTAACTCTGGTCACATAT
*ITGB1*	Integrin aubunit beta 1	3688	Forward	ACAGTGAAGACATGGATGCTTACT
			Reverse	ACGACACTTGCAAACACCATTTC
*FAK*	Focal adhesion kinase	5747	Forward	CATCTATCCAGGTCAGGCATCTC
			Reverse	TTTCCTGTTGCTGTCGGATTAGA
*TPM1*	Tropomyosin 1	7168	Forward	GTTTGCGGAGAGGTCAGTAACTA
			Reverse	TCAGGGCCAGCTTTAGTTCATTA
*ACTB*	Actin beta	60	Forward	ACAGAGCCTCGCCTTTGC
			Reverse	CCACCATCACGCCCTGG
*ACTN1*	Actinin alpha 1	87	Forward	TCAACAAGGCCCTGGATTTCATA
			Reverse	TGTGGAAGTTCTGGATGTTGACA
*RAC1*	Rac family small GTPase 1	5879	Forward	GGCTAAGGAGATTGGTGCTGT
			Reverse	GACAGGACCAAGAACGAGGG
*RHOA*	Ras homolog family member A	387	Forward	TTCGTTGCCTGAGCAATGG
			Reverse	TGTGTCCCACAAAGCCAACT

### Immunofluorescence Staining for Fibrogenic Differentiation Analysis

Fibrogenic differentiation‐related markers (SCX and COL‐1) were detected by immunofluorescence staining. Cells were rinsed three times with PBS and then fixed in 4% paraformaldehyde (Servicebio) for 15 min. After washing three times with PBS, cells were permeabilized with 0.1% Triton X‐100 (MP Biomedical) at room temperature for 5 min. Then, the cells were rinsed and incubated at 4 °C overnight with the following primary antibodies: rabbit anti‐SCXA (1:100, DF13293, Affinity Biosciences, Cincinnati, OH, USA) and rabbit anti‐COL‐1 (1:200, 72026, Cell Signaling Technology, Boston, MA, USA). Subsequently, the cells were rinsed and incubated in Alexa Fluor 488 donkey anti‐rabbit IgG (H+L) (1:500, 34206ES60, Yeasen) plus DAPI (1:1000, 40728ES03, Yeasen) for 1 h at room temperature in the dark. Representative images were captured by confocal microscopy (Nikon) at 20× magnification with the same settings.

### Sirius Red Total Collagen Detection

The content of total collagen in the supernatant was detected by a Sirius red total collagen detection kit (cat. 9062, Chondrex, Washington, WV, USA) following the manufacturer's instructions. In brief, 1 mL of collected supernatant was mixed with 250 µL of concentrating solution, and the mixture was vortexed overnight at 4 °C. Subsequently, each sample was centrifuged at 10 000 rpm for 3 min, and 250 µL of 0.05 m acetic acid was added to each centrifuge tube to dissolve the obtained pellet. After complete dissolution, 100 µL of solution was mixed with 500 µL of Sirius Red in another 1.5‐mL centrifuge tube. After vortexing for 20 min at room temperature, the mixture was centrifuged, rinsed in 500 µL of Washing Solution, and centrifuged at 10 000 rpm for 3 min. Then, 250 µL of Extraction Buffer was added to dissolve the precipitate. After vortexing and complete dissolution, 200 µL of solution from each tube was transferred to a 96‐well plate. The optical density (OD) value at 510 nm was measured using a microplate reader (Tecan). The concentrations of collagen in each sample were calculated from standard curves, which were defined by the OD values and corresponding concentrations (125, 63, 31.5, 16, and 8 µg mL^−1^).

### The Mechanical Responses of PDLSCs to Viscoelastic F127DA Hydrogels under Cytomechanical Loading

Given that integrin–FAK–cytoskeleton pathways play key roles in cell–ECM interactions, which influence the spreading and differentiation of stem cells by activating mechanoresponsive signaling and mechanotransduction pathways,^[^ ^49^, ^62^ ^]^ the cell spreading of PDLSCs and the related integrin–FAK–cytoskeleton pathways were further analyzed. As described before, PDLSCs were seeded on the surfaces of viscoelastic F127DA hydrogels and stimulated with compressive pressure (≈0–120 kPa, 1 Hz, 1 h day^−1^). After 24 h of cytomechanical loading, the morphology of PDLSCs cultured on F127DA‐5, F127DA‐10, or F127DA‐20 hydrogels was observed by optical microscope, phalloidin staining, and SEM according to the methods described and focal adhension‐related markers (Vinculin and Talin) were detected by immunofluorescent staining, the following primary antibodies were used: rabbit anti‐Talin (1:200, 14168‐1‐AP, Proteintech, Wuhan, China) and mouse anti‐Vinculin (1:200, 66305‐1‐Ig, Proteintech). Subsequently, the cells were rinsed and incubated in Alexa Fluor 488 Goat anti‐Mouse IgG (H+L) (1:500, 33206ES60, Yeasen), Alexa Fluor 594 Goat anti‐Rabbit IgG (H+L) (1:500, 33112ES60, Yeasen) plus DAPI (1:1000, 40728ES03, Yeasen) for 1 h at room temperature in the dark. The elastic modulus of PDLSCs cultured on F127DA‐5, F127DA‐10, or F127DA‐20 hydrogels was detected by AFM single‐cell experiments according to the aforementioned methods. The expression levels of integrin–FAK‐cytoskeleton pathway‐related genes (*ITGA5, ITGB1, FAK, ACTN1, ACTB, TPM1, RAC1*, and *RhoA*) were analyzed by qRT–PCR. The primer sequences used in this study are listed in Table 1.

### Influence of Cytochalasin B Treatment on the Mechanical Responses and Fibrogenic Differentiation of PDLSCs under Cytomechanical Loading

To further examine the effect of the cytoskeleton on the fibrogenic induction of PDLSCs, cells cultured on F127DA‐5 hydrogels were first incubated in complete medium containing 10 µM cytochalasin B (CB; Yeasen) for 24 h under cytomechanical loading. Then, the spreading of PDLSCs was observed by optical microscopy and phalloidin staining. In line with the cell spreading analysis, the expression levels of integrin–FAK–cytoskeleton pathway‐related genes (ITGA5, ITGB1, FAK, ACTN1, and TPM1) were analyzed by qRT‒PCR according to the aforementioned methods. Similarly, the fibrogenic differentiation potential of PDLSCs was determined using qRT‒PCR and immunofluorescence staining after cells cultured on F127DA‐5 hydrogels were incubated in fibrogenic inductive medium containing CB for 7 days.

### RNA Sequencing Analysis

To further understand the underlying mechanics of Viscoelastic F127DA hydrogels enhanced the fibrogenic differentiation potentials of PDLSCs under cytomechanical loading. RNA sequencing analysis was performed to obtain a global view of the affected biological processes and signaling pathways within PDLSCs. The samples were divided into three groups, including F127DA‐5 group, F127DA‐10 group, and F127DA‐20 group, with 3–4 parallel samples in each group. After an incubation period of 7 days, the PDLSCs cultured on F127DA‐5, F127DA‐10, or F127DA‐20 hydrogels were harvested for RNA sequencing analysis, the Trizol was used to lysis cells and total RNA was extracted by a total RNA kit. All samples were submitted to Biomarker Technologies Co., Ltd. (Beijing, China). After extracting the total RNA, a library was constructed to check and ensure the quality of RNA, and then Illumina sequencing was carried out. After sequencing, all the genes were analyzed statistically, and the differentially expressed genes (DEGs) in different groups were compared. The screening criterion of differential genes was log2 (fold change) ≥ 1.5, and *p*‐value ≤ 0.05. Then the Gene Ontology (GO), Kyoto Encyclopedia of Genes and Genomes (KEGG) pathway enrichment analysis, and gene set enrichment analysis (GSEA) were performed and visualized based on differentially expressed genes DEGs using the BMKCloud (www.biocloud.net).

### In Vivo Biodegradability of F127DA Hydrogels

The biodegradation of F127DA hydrogels following dorsal subcutaneous transplantation was analyzed using in vivo immunofluorescence images. Nine male C57 mice aged 7–8 weeks (purchased from the Laboratory Animal Center of FMMU) were used in the experiment. All surgical procedures were approved by the Animal Research Committee of FMMU (grant no. IACUC‐20240112). F127DA hydrogel solutions were first labeled with the fluorescent dye (4 mg mL^−1^; EFL‐DYE‐UF‐ENE‐R, EFL) following the manufacturer's instructions. Then the F127DA solutions were added into 2‐mL syringes to fabricate cylindrical F127DA hydrogel samples (3 mm in height). F127DA hydrogel samples were then subcutaneously transplanted into the bilateral backs of mice. The fluorescent dye‐labeled F127DA hydrogels were detected using the In Vivo Imaging System (IVIS) (Xenogen, Alameda, CA, USA) at 0, 1, and 2 weeks post surgery. The radiant efficiency was quantified using Living Image v.4.3.1 software (Caliper Life Sciences, Hopkinton, MA, USA). The following formula was used to calculate the in vivo retention (%) of F127DA hydrogels according to a previously published study.

(4) $$\mathrm{Retention}\;\mathrm{of}\;F127\mathrm{DA}\;\left(\%\right)=\left[RE\left(t\right)\right]/\left[RE\left(t_{0}\right)\right]\times 100\%$$

where *RE(t*_0_) is the radiant efficiency at week 0 and *RE(t)* is the radiant efficiency at time point *t*.

### Therapeutic Performance of Viscoelastic F127DA Hydrogels on PDL Injury in Delayed Replantation of Avulsed Teeth

A model of delayed replantation of an avulsed incisor was used as a proof‐of‐concept test to evaluate the effects of viscoelastic F127DA hydrogels on PDL regeneration. Twenty‐four male Sprague−Dawley rats (7–8 weeks, 180–250 g; purchased from the Laboratory Animal Center of FMMU) were used for delayed tooth replantation, in accordance with the ethical guidelines. All surgical procedures were also approved by the Animal Research Committee of FMMU (grant no. IACUC‐20221241). Based on previously published methods,^[^ ^75^, ^86^ ^]^ animals were anesthetized by the intraperitoneal injection of 0.1% pentobarbital sodium (0.4 mL/100 g). After general anesthesia, the maxillary left incisors of rats were submucosally anesthetized using primacaine (Acteon Pharma, Bordeaux, Gironde, France), and then the maxillary left incisor of each rat was completely extracted with minimum trauma using a 1# Root Elevator (BONEWELL Medical, Suzhou, Jiangsu, China). Teeth with either root or bone fracture were excluded from the present study. Immediately after extraction, the dental papilla and pulp tissues were carefully removed using a 00# barbed broach (MANI, Tokyo, Japan) to prevent the continual growth of the rat incisors. The root canals were then irrigated with sterile 0.9% physiological saline, and the residual saline was dried by #40 absorbent paper points (HuaYou Medical Instruments, Ziyang, Sichuan, China). To prevent potential infection, the root canal was filled with calcium hydroxide root canal filling agent (C‐Root, Beijing, China). Next, the dental crowns of extracted teeth were inserted in an orthodontic wax, and then the tooth roots were air‐dried in a fume hood for 60 min. Thereafter, each root surface was packaged with 20 µL of F127DA‐5, F127DA‐10, or F127DA‐20 hydrogel. Tooth roots without hydrogel coatings were used as a control. After crosslinking under 405 nm UV‐light exposure for 30 s, each tooth was replanted into the corresponding extraction socket with slow and delicate movements using tweezers. Penicillin was intramuscularly injected, and iodine tincture was locally applied for 3 consecutive days to prevent potential postoperative infection. The rats were fed a soft food diet for 7 days and then given a regular diet. At 8 weeks post surgery, the animals were euthanized by CO_2_, and the maxillaries were harvested for further analysis.

### Micro‐CT Analysis

The maxillaries were collected and fixed in 4% paraformaldehyde (Servicebio) for 24 h. Then, root resorption and PDL regeneration were analyzed using a micro‐CT scanner (AX2000, Always Imaging, Shanghai, China) with a resolution of 12.5 µm, an energy of 90 kV, and a current of 80 µA. After scanning, the obtained images were analyzed and three‐dimensionally reconstructed using the software VG studio MAX 3.5.1 (Volume Graphics, Heidelberg, Baden‐Württemberg, Germany). After the 3D reconstruction of rat maxillary incisors by software, the degree of root resorption was calculated as the root resorption area/total root area (100%) measured by the software. Since the PDL of rat incisors exists only on the lingual side,^[^ ^87^ ^]^ the region of interest (ROI) was defined as the lingual area between the root and alveolar bone to reconstruct the PDL model. The ROI of each sample was analyzed to determine the area (mm^2^) and volume (mm^3^) of reconstructed PDL tissue.

### Histology Analysis

The newly formed periodontal tissue and root resorption were analyzed based on Masson and H&E staining according to the previously published methods.^[^ ^88^ ^]^ Briefly, after micro‐CT scanning, the specimens were decalcified in 10% EDTA decalcification solution (Coolaber, Beijing, China) at 37 °C for at least 3 months. The decalcification solution was exchanged every 3 days. Subsequently, the obtained samples were dehydrated and embedded in paraffin and sectioned longitudinally along the entire length of the tooth from crown to root at a thickness of 4 µm. The slices were further stained with Masson and H&E. The scanned slices were observed using slide‐viewing software (CaseViewer ver. 2.1, 3DHISTECH). The histomorphometric analysis included the following parameters: root resorption, replacement root resorption (ankylosis), and the length of the newly formed PDLs.

### Immunofluorescence Staining for Newly Regenerated PDL Evaluation

For immunofluorescence staining, sections were routinely dewaxed and hydrated, and then the antigen in specimens was retrieved using sodium citrate buffer (DIYIBio). Subsequently, sections were blocked and incubated in the following primary antibodies at 4 °C overnight: rabbit anti‐COL‐1 (1:100, 14695‐1‐AP, Proteintech) and rabbit anti‐SCX (1:300, bs‐12364R, Bioss, Beijing, China). Subsequently, the slices were rinsed with PBS and incubated with the following two secondary antibodies: Alexa Fluor 488 donkey anti‐rabbit IgG (1:500; 34206ES60, Yeasen) or Alexa Fluor 594 donkey anti‐rabbit IgG (1:500, 34212ES60, Yeasen) plus DAPI (1:1000, 40728ES03, Yeasen). After rinsing with PBS three times, the images were scanned by slide‐viewing software (CaseViewer ver. 2.1, 3DHISTECH).

### Statistical Analysis

Data from at least three independent experiments are presented as the means ± standard deviations. When normality was confirmed, one‐ or two‐way ANOVA with Tukey's post hoc test was performed to evaluate the group differences when dealing with more than 2 groups. The statistical significance of the difference between two groups was determined by unpaired *t*‐test. When the data were nonnormally distributed, the Kruskal‒Wallis test was used to calculate statistical significance. In all cases, significance was defined as follows: *p < 0.05, **p < 0.01, ***p < 0.001. The *n* values in each figure legend represent the number of the sample size or repetitions. Detailed *P* values, *n* values, and statistical methods were listed in the corresponding figure legends.

## Conflict of Interest

The authors declare no conflict of interest.

## Author Contributions

J.‐J.Z., X.L., and Y.T. contributed equally to this work. R.‐X.W., F.‐M.C., and X.‐T.H. conceptualized the study and designed the experiments. J.‐J.Z., X.L., and X.‐T.H. supervised the study. J.‐J.Z. and Y.T. synthesized the materials. J.‐J.Z., X.L., and Y.T. characterized the samples. J.‐J.Z., X.L., J.‐K.Z., and D.‐K.D. performed the in vitro experiments. J.‐J.Z., Y.T., and C.J. performed the in vivo experiments. X.L., Y.Y., B.‐M.T., and X.‐T.H. analyzed the data. J.‐J.Z., X.L., Y.T., and X.‐T.H. wrote and drafted the manuscript.

## Supporting information

Supporting Information

## Data Availability

The data that support the findings of this study are available from the corresponding author upon reasonable request.
