# Supplementary material for: Harnessing Mechanical Stress with Viscoelastic Biomaterials for Periodontal Ligament Regeneration
Source: Adv Sci (Weinh). 2024 Mar 9;11(18):2309562. doi: 10.1002/advs.202309562 (PMC11095218; doi:10.1002/advs.202309562)
Supplement: Supplementary file 1 — Supporting Information [file ADVS-11-2309562-s001.pdf]

ADVANCED SCIENCE
Open Access

# Supporting Information

for *Adv. Sci.*, DOI 10.1002/advs.202309562

Harnessing Mechanical Stress with Viscoelastic Biomaterials for Periodontal Ligament Regeneration

*Jiu-Jiu Zhang, Xuan Li, Yi Tian, Jie-Kang Zou, Dian Gan, Dao-Kun Deng, Chen Jiao, Yuan Yin, Bei-Min Tian, Rui-Xin Wu*, Fa-Ming Chen* and Xiao-Tao He**

# Harnessing Mechanical Stress with Viscoelastic Biomaterials for Periodontal Ligament Regeneration

J.-J. Zhang, X. Li, Y. Tian, J.-K. Zou, D. Gan, D.-K. Deng, C. Jiao, Y. Yin, B.-M. Tian, R.-X. Wu*, F.-M. Chen*, X.-T. He*

State Key Laboratory of Oral & Maxillofacial Reconstruction and Regeneration, National Clinical Research Center for Oral Diseases, Shaanxi International Joint Research Center for Oral Diseases, Department of Periodontology, School of Stomatology, The Fourth Military Medical University, Xi'an, 710032, China

E-mail: hexiao_tao@163.com, cfmsunhh@fmmu.edu.cn, gracia-wu@foxmail.com

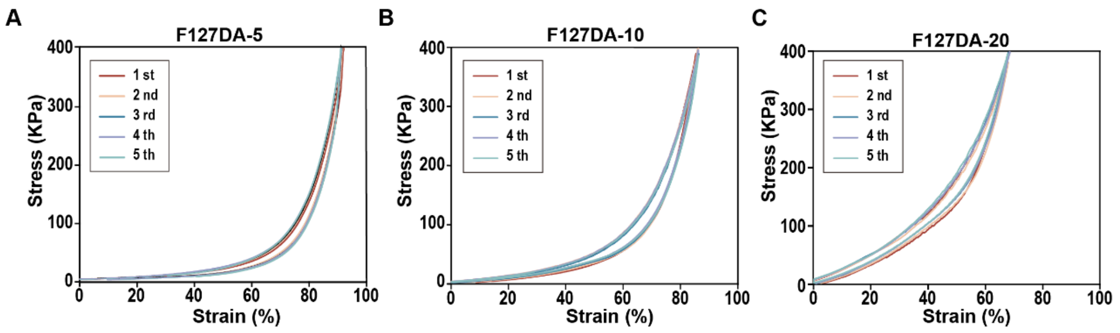


**Figure S1.** Mechanical stability of F127DA-5 (**A**), F127DA-10 (**B**) and F127DA-20 (**C**) hydrogels during 5 cycles of loading-unloading compression tests.

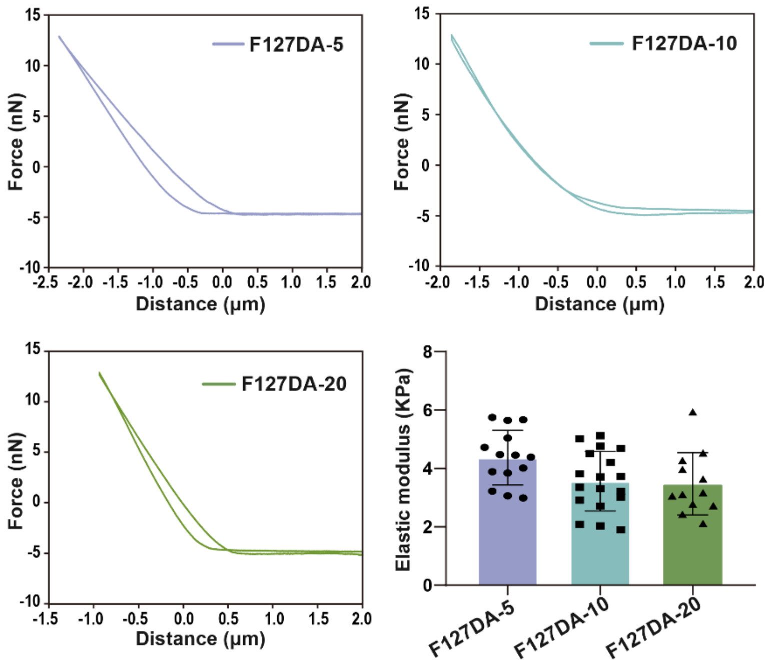


**Figure S2.** Representative force-distance curves and calculated elastic modulus of PDLSCs cultured on F127DA-5, F127DA-10 and F127DA-20 hydrogels under static conditions (Atomic force microscopy assay) ($n$ = 12-19). The data are shown as the mean ± SD. Data shown in Figure S2 were analyzed by one-way ANOVA; no significant differences could be observed.

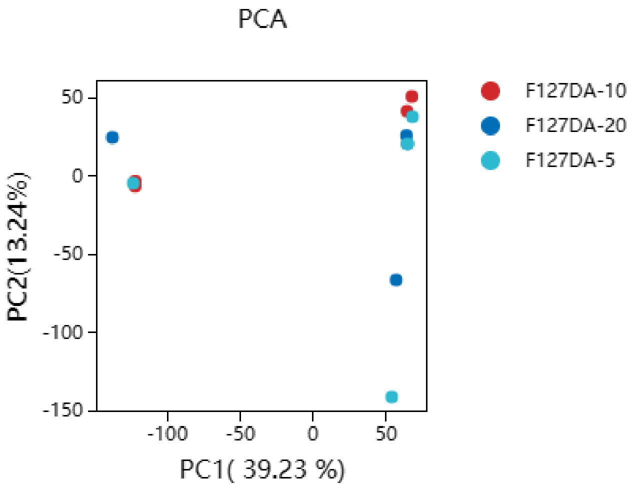


**Figure S3.** Principal component analysis (PCA) plot of RNA seq data in F127DA-5, F127DA-10 and F127DA-20 groups under static condition. Four samples were measured per group.

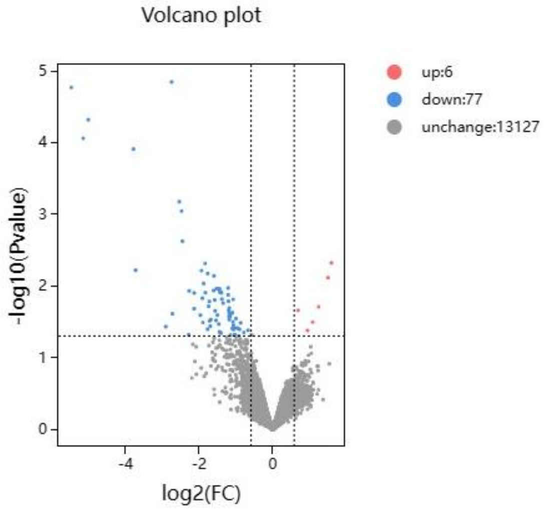


**Figure S4.** Volcano plot of all gene expressions in F127DA-5 vs. F127DA-10 groups under static condition. Four samples were measured per group.

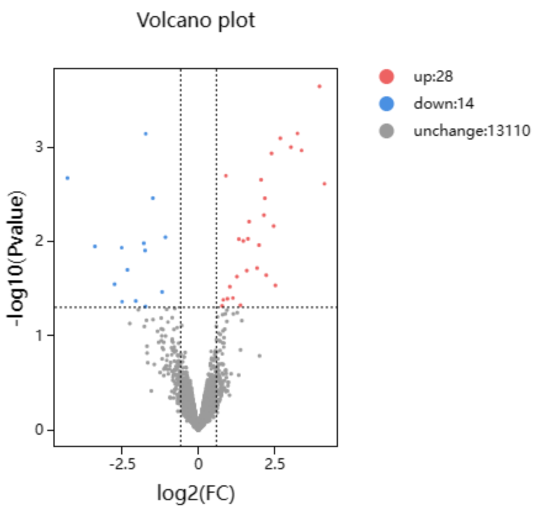


**Figure S5.** Volcano plot of all gene expressions in F127DA-5 vs. F127DA-20 groups under static condition. Four samples were measured per group.

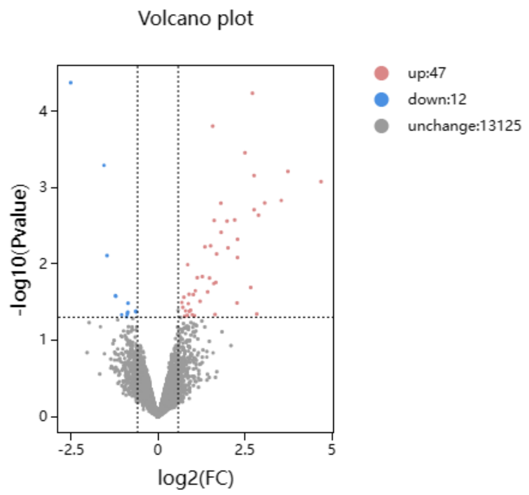


**Figure S6.** Volcano plot of all gene expressions in F127DA-10 vs. F127DA-20 groups under static condition. Four samples were measured per group.

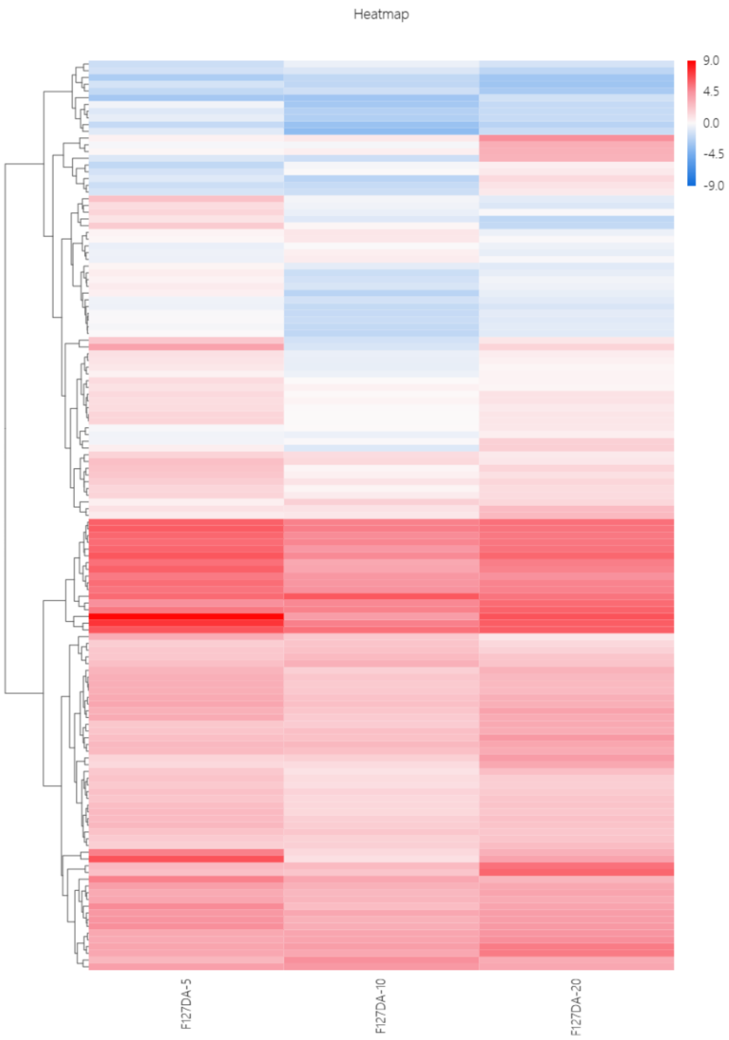


**Figure S7.** Heat map of all identified gene expressions in F127DA-5, F127DA-10 and F127DA-20 groups under static condition after cluster analysis. Four samples were measured per group.

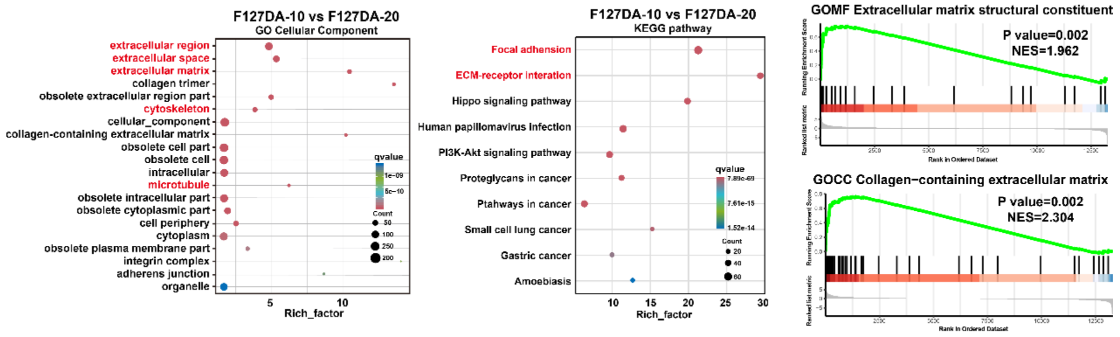

**Figure S7.** GOCC, KEGG and GSEA analysis of DEGs in F127DA-10 vs. F127DA-20 under cytomechanical loading. Three to four samples were measured per group.

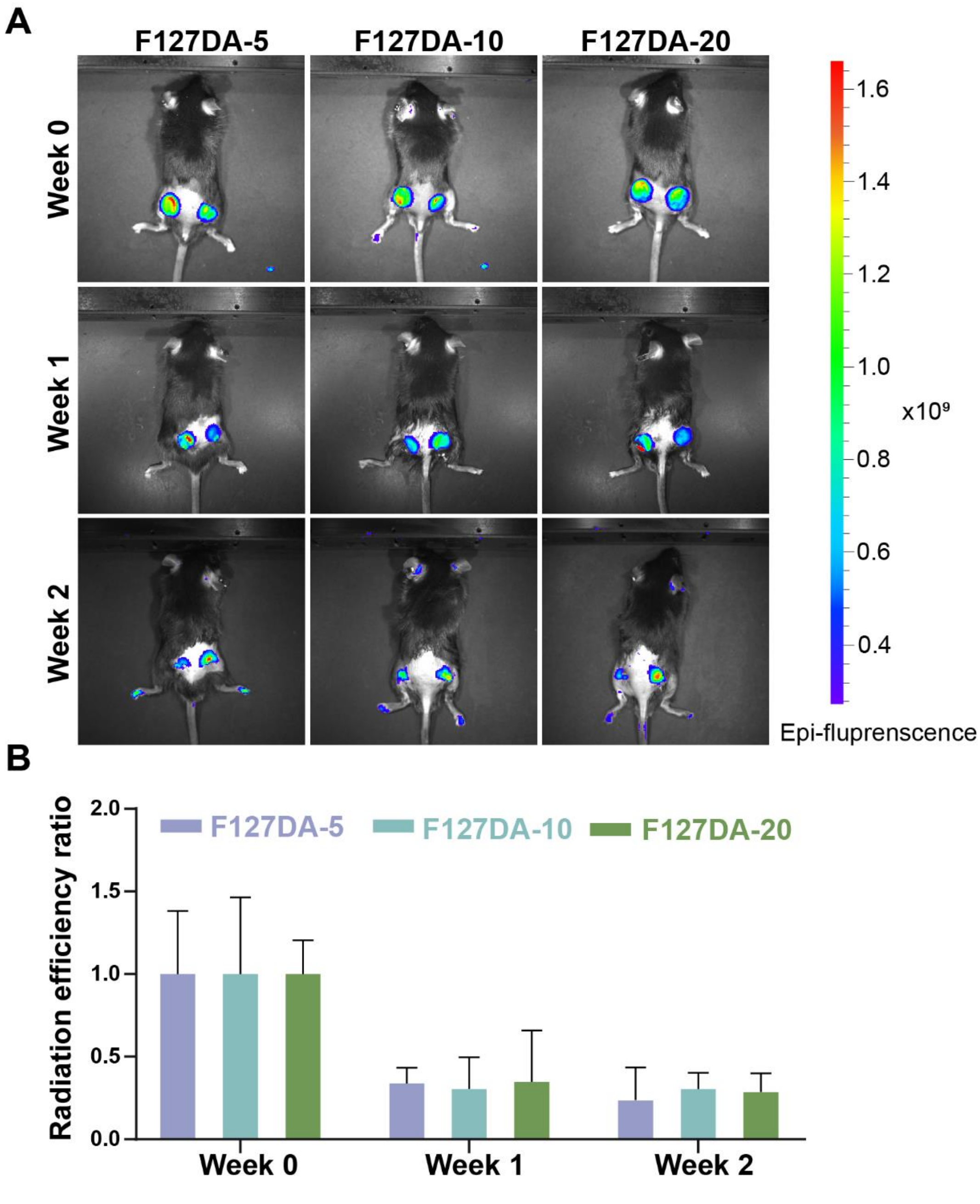


**Figure S8.** The biodegradation of F127DA-5, F127DA-10 and F127DA-20 hydrogels. (**A**) Representative in vivo fluorescence images showing the retention of F127DA hydrogels at 1 and 2 weeks following subcutaneous implantation. (**B**) Quantitative analysis of F127DA retention by calculating the radiation efficiency ratio (*n* = 3-4). The data are shown as the mean ± SD. Data shown in (B) were analyzed by two-way ANOVA; no significant differences could be observed.
